# Strain-dependent modifiers exacerbate familial leukemia caused by GATA1-deficiency

**DOI:** 10.1186/s40164-024-00491-w

**Published:** 2024-02-26

**Authors:** Ikuo Hirano, Kanako Abe, James Douglas Engel, Masayuki Yamamoto, Ritsuko Shimizu

**Affiliations:** 1 Department of Molecular Hematology, Tohoku University Graduate School of Medicine, 2-1 Seiryo-machi, Aoba-ku, Sendai, 980-8575 Miyagi Japan; 2 Tohoku Medical Megabank Organization, Tohoku University, Sendai, 980-8575 Miyagi Japan; 3 Ibaraki Prefectural Central Hospital, Ibaraki Cancer Center, Ibaraki, 309-1793 Japan; 4 Department of Cell and Developmental Biology, University of Michigan Medical School, Ann Arbor, MI 48109 USA

**Keywords:** Erythroid, GATA1 transcription factor, Leukemia, Modifier, Transformation

## Abstract

GATA1 plays a critical role in differentiation, proliferation, and apoptosis during erythropoiesis. We developed a *Gata1* knock-down allele (*Gata1.05*) that results in GATA1 expression at 5% of endogenous level. In female mice heterozygous for both the *Gata1.05* and wild-type alleles, we observed a predisposition to erythroblastic leukemia three to six months after birth. Since no male *Gata1.05* progeny survive gestation, we originally maintained heterozygous females in a mixed genetic background of C57BL/6J and DBA/2 strains. Around 30% of these mice reproducibly develop leukemia, but the other subset did not develop leukemia, even though they harbor a high number of preleukemic erythroblasts. These observations prompted us to hypothesize that there may be potential influence of genetic determinants on the progression of *Gata1.05*-driven hematopoietic precursors to full-blown leukemia. In an initial examination of *Gata1.05*/X mice backcrossed into C3H/He, BALB/c, DBA/2, C57BL/6J and 129X1/SvJ strains, we discerned that the backgrounds of C57BL/6J and 129X1/SvJ significantly expedited leukemia onset in *Gata1.05*/X mice. Conversely, backgrounds of C3H/He, BALB/c and DBA/2 did not substantially modify the effect of the *Gata1* mutation. This indicates the existence of genetic modifiers that accentuate *Gata1.05* leukemogenesis. Subsequent cohort studies evaluated *Gata1.05*/X mice within mix backgrounds of BALB/c:129X1/SvJ and BALB/c:C57BL/6J. In these settings, *Gata1.05*-driven leukemia manifested in autosomal dominant patterns within the 129X1/SvJ background and in autosomal recessive patterns within C57BL/6J background. To the best of our knowledge, this study provides the inaugural evidence of genetic modifiers that can reshape the outcome based on leukemia-associated gene signatures.

**Supplementary Information:**

The online version contains supplementary material available at 10.1186/s40164-024-00491-w.

To the editor,

GATA1 is a transcription factor that plays crucial roles in erythropoiesis, megakaryopoiesis, and other hematopoietic pathways [1–4]. We previously established a *Gata1.05* allele, in which *Gata1* gene expression is reduced into 5% of endogenous level [5]. Since the *Gata1* gene is located on the X-chromosome, heterozygous female mice (*G1*^*1.05*^) carry two types of erythroid progenitors due to random X-chromosome inactivation. Erythroid progenitors with an active *Gata1.05* allele and an inactive wild-type X-chromosome struggle to differentiate into matured cells, leading to the accumulation of immature erythroid progenitors in the *G1*^*1.05*^ [6–9]. These abnormal erythroid progenitors frequently undergo cancerous changes. Consequently, approximately 30% of *G1*^*1.05*^ in a mixed background colony of C57BL/6J:DBA/2 strains are predisposed to develop erythroleukemia, which morphologically resembles human pure erythroid leukemia [6–10]. These findings have led us to hypothesize that genetic variations exist between the C57BL/6J and DBA/2 strains, which may act as modifiers of *Gata1.05*-driven leukemogenesis.

To investigate this hypothesis, we generated a mouse cohort of 369 *G1*^*1.05*^. These mice were produced by backcrossing *G1*^*1.05*^ in a mixed background to five inbred strains: C3H/He, BALB/c, DBA/2, C57BL/6J, and 129X1/SvJ. The resulting strains were named C3.*G1*^*1.05*^, C.*G1*^*1.05*^, D2.*G1*^*1.05*^, B6.*G1*^*1.05*^, 129.*G1*^*1.05*^, respectively, followed by the number of backcross generations. Eventually, we used 44 mice of C.*G1*^*1.05*^ (N2-N9), 20 mice of C3.*G1*^*1.05*^ (N2-N6), 126 mice of B6.*G1*^*1.05*^ (N2-N8), 40 mice of D2.*G1*^*1.05*^ (N2-N6), and 139 mice of 129.*G1*^*1.05*^ (N2-N9) for the survival time analysis. We then studied the development of leukemia in these mice. Our findings revealed a significantly elevated risk of early mortality in B6.*G1*^*1.05*^ and 129.*G1*^*1.05*^ when compared to the other three backgrounds (Fig. 1A). In both the B6.*G1*^*1.05*^ and 129.*G1*^*1.05*^ groups, the peak mortality occurred between 90 and 180 days of age. This aligns closely with a previous report on leukemogenesis in *G1*^*1.05*^ in a mixed background [6].

**Fig. 1:**
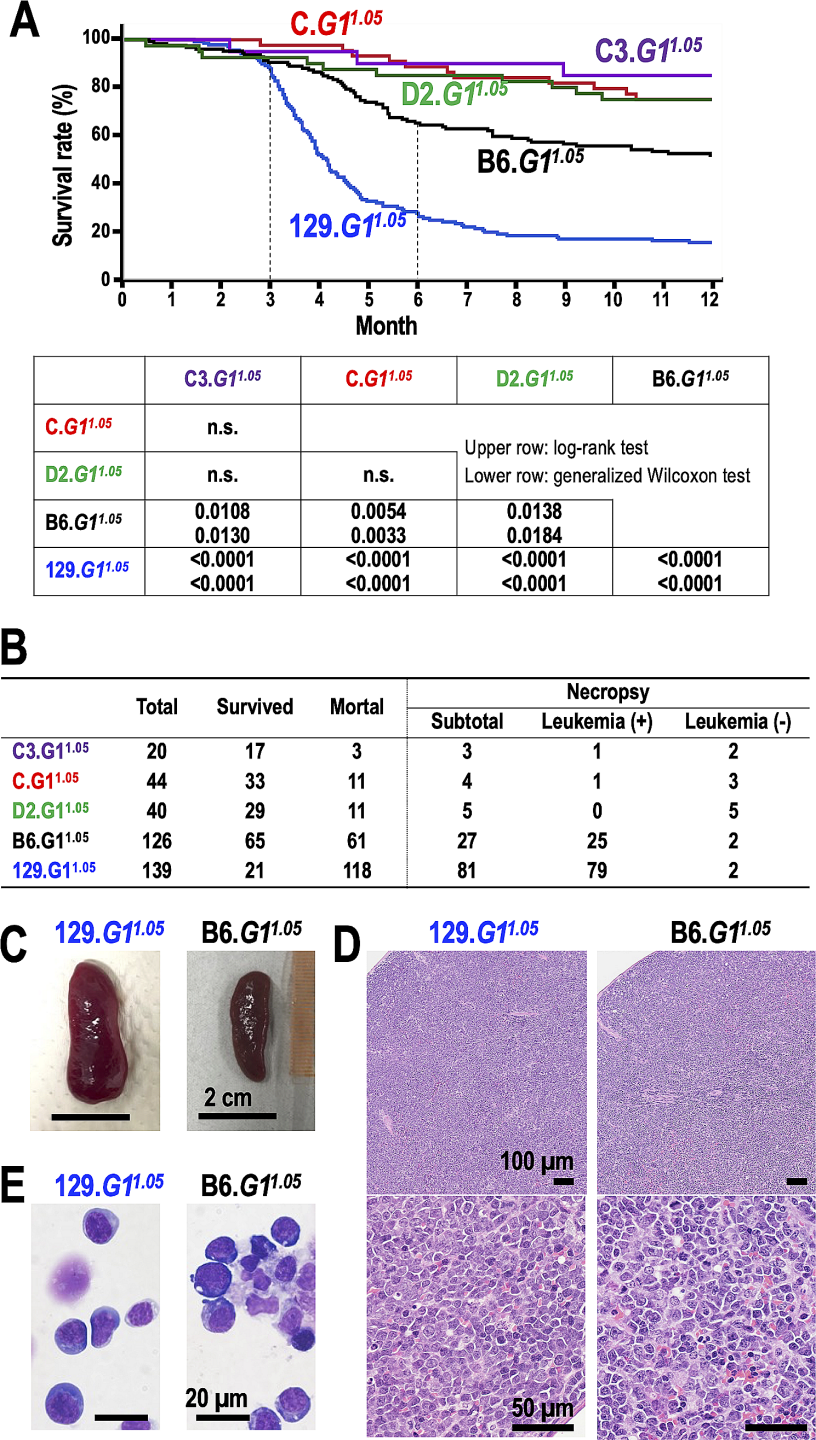
Mortality of *Gata1.05*/X mice (*G1*^*1.05*^) is affected by genetic backgrounds. **A** Kaplan-Meier curves depicting the overall survival of *G1*^*1.05*^ in C57BL/6J (black line, *n* = 126), 129X1/SvJ (blue line, *n* = 139), BALB/c (red line, *n* = 44), DBA/2 (green line, *n* = 40) and C3H (purple line, *n* = 20) backgrounds. Mice that were backcrossed onto the indicated inbred strains twice to nine times were used in this experiment. The details of mice used in this experiment are provided in Supplementary Table 1. A summary of the Log-rank test (upper row) and Generalized Wilcoxson test (lower row) results between the indicated groups is shown in table. n.s; not significant. **B** Observed mortality of C.*G1*^*1.05*^, C3.*G1*^*1.05*^, B6.*G1*^*1.05*^, D2.*G1*^*1.05*^ and 129.*G1*^*1.05*^, along with their necropsy results. **C-E** Leukemia phenotype in B6.*G1*^*1.05*^ and 129.*G1*^*1.05*^ that underwent necropsy. Representative images of the macroscopic (**C**), Hematoxylin-Eosin microscopic (**D**), and May-Giemsa-stained cytospin (**E**) analyses of spleens from *G1*^*1.05*^ developing leukemia in 129X1/SvJ (left) and C57BL/6J (right) backgrounds. Note that leukemia phenotypes of mice in 129X1/SvJ and C57BL/6J backgrounds do not exhibit substantial differences

Necropsy analyses revealed a strong correlation between leukemogenesis and mortality in B6.*G1*^*1.05*^ and 129.*G1*^*1.05*^ (Fig. 1B). These analyses showed pronounced splenomegaly characterized by a destroyed splenic structure due to the extensive invasion of proerythroblast-like leukemic cells (Fig. 1C-E). The leukemic alterations in 129.*G1*^*1.05*^ (Fig. 1C-E; left panels) and B6.*G1*^*1.05*^ (right panels) were indistinguishable between and bore resemblance to those observed in mice in a mixed background [6]. In contrast, when we examined four C.*G1*^*1.05*^, three C3.*G1*^*1.05*^, and five D2.*G1*^*1.05*^ animals euthanized within a year as a humane endpoint, only two mice were found to have developed leukemia (Fig. 1B). Thus, the penetrance of *Gata1.05*-driven leukemogenesis appears to be strongly influenced by the genetic backgrounds of the mice.

To investigate the leukemia susceptibility phenotype of 129.*G1*^*1.05*^ and B6.*G1*^*1.05*^, we established two validation mouse cohorts by crossing C.*G1*^*1.05*^, which rarely develop leukemia, with wild-type 129X1/SvJ or C57BL/6J males. We then crossbred the resulting F1 generation mice (F1-C:129.*G1*^*1.05*^ and F1-C:B6.*G1*^*1.05*^, respectively) to produce the second generation (Fig. 2A,B). Interestingly, F1-C:129.*G1*^*1.05*^ exhibited a high mortality rate, with over half not surviving past six months (Fig. 2C). Comparable survival curves were observed in both N2-129.*G1*^*1.05*^ and F2-C:129.*G1*^*1.05*^ (Fig. 2A). The former represents the second backcross generation produced by crossing F1-C:129.*G1*^*1.05*^ with 129X1/SvJ inbred males, while the latter denote the second filial generation produced by brother and sister intercross of F1-C:129.*G1*^*1.05*^. Notably, F2-C:129.*G1*^*1.05*^ showed a modestly increased survival rate when compared to F1-C:129.*G1*^*1.05*^ or N2-129.*G1*^*1.05*^ (Fig. 2C). When comparing the survival of F1-C:129.*G1*^*1.05*^, N2-129.*G1*^*1.05*^ and F2-C:129.*G1*^*1.05*^ with 129.*G*^*1.05*^ used in the discovery cohort study, we found that the survival rates of F1-C:129.*G1*^*1.05*^ and N2-129.*G1*^*1.05*^ were almost similar to that of 129.*G*^*1.05*^, while the survival rate of F2-C:129.*G1*^*1.05*^ was significantly increased (Supplementary Fig. 1A). Given that a quarter of the F2-C:129.*G1*^*1.05*^ cohort were BALB/c progeny, it seems plausible that these mice contributed to the slightly enhanced survival rate, especially as they infrequently developed leukemia.

**Fig. 2:**
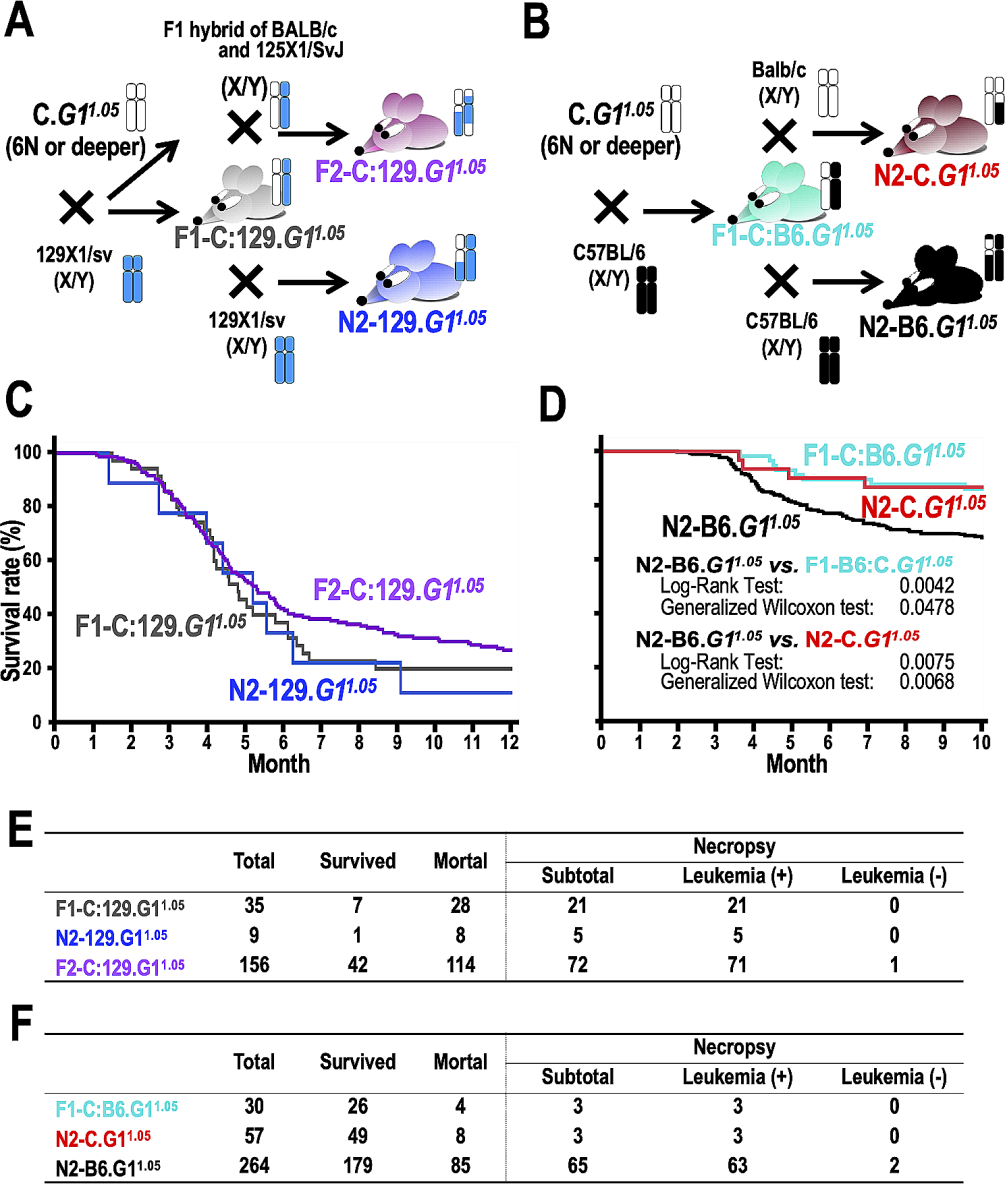
Inherited susceptibility to leukemia in *Gata1.05*/X mice (*G1*^*1.05*^) in 129X1/SvJ and C57BL/6J backgrounds. **A** Schematic illustration of mouse mating strategy. N6 or deeper generation C.*G1*^*1.05*^ mice were prepared by backcrossing *G1*^*1.05*^ in a mixed background with BALB/c mice six and more generations. These C.*G1*^*1.05*^ were crossed with 129X1/SvJ wild-type males, and subsequently F1-C:129.*G1*^*1.05*^ (shown in gray) were generated. Note that F1-C:129.*G1*^*1.05*^ display heterozygosity at the *Gata1* locus (i.e., *Gata1.05* and wild-type alleles), with equal contributions from the BALB/c and 129X1/SvJ backgrounds on all autosomes. *G1*^*1.05*^ generated by crossing F1-C:129.*G1*^*1.05*^ with wild-type 129X1/SvJ males are defined as N2-129.*G1*^*1.05*^ (shown in blue). We also generated F2-C:129.*G1*^*1.05*^ (shown in purple) through brother-sister crossing of F1-C:129.*G1*^*1.05*^ and F1-C:129 males. Thus, N2-129.*G1*^*1.05*^ (blue) harbor either a homozygous 129X1/SvJ background or a heterozygous BALB/c:129X1/SvJ background. Similarly, F2-C:129.*G1*^*1.05*^ (purple) harbor a homozygous BALB/c background, a homozygous 129X1/SvJ background, or a heterozygous BALB/c:129X1/SvJ background. Details of the mice examined are provided in Supplementary Table 2B Schematic illustration of mouse mating strategy. N6 or deeper generation C.*G1*^*1.05*^ mice were used. *G1*^*1.05*^ obtained by crossing C.*G1*^*1.05*^ with wild-type C57BL/6J males are defined as F1-C:B6.*G1*^*1.05*^ (shown in turquois). Crossing F1-C:B6.*G1*^*1.05*^ with wild-type BALB/c and C57BL/6J males generated N2-C.*G1*^*1.05*^ (shown in red) and N2-B6.*G1*^*1.05*^ (shown in black), respectively. Note that F1-C:B6.*G1*^*1.05*^ display heterozygosity at the *Gata1* locus (i.e., *Gata1.05* and wild-type alleles), with equal contributions from the BALB/c and C57BL/6J backgrounds on all autosomes. N2-C.*G1*^*1.05*^ (red) harbor a BALB/c:C57BL/6J heterozygous background or a BALB/c homozygous background, and N2-B6.*G1*^*1.05*^ (black) harbor a BALB/c:C57BL/6J heterozygous background or a C57BL/6J homozygous background. Details of the mice examined are provided in Supplementary Table 3C Kaplan-Meier curves of the overall survival of F1 C:129.*G1*^*1.05*^ (gray line, *n* = 35), N2 129.*G1*^*1.05*^ (blue line, *n* = 9), and F2C:129.*G1*^*1.05*^ (purple line, *n* = 156). **D** Kaplan-Meier curves of the overall survival of F1-C:B6.*G1*^*1.05*^ (turquois line, *n* = 30), N2-C.*G1*^*1.05*^ (red line, *n* = 57), and N2-B6.*G1*^*1.05*^ (black line, *n* = 264). Results of Log-rank tests and Generalized Wilcoxson tests in comparison of N2-B6.*G1*^*1.05*^ vs. F1-B6:C.*G1*^*1.05*^ and N2-B6.*G1*^*1.05*^ vs. N2-C.*G1*^*1.05*^ are indicated. **E** Observed mortality data for mice used in **C** and necropsy results. **F** Observed mortality data for mice used in **D** and necropsy results

In stark contrast, over 80% of F1-C:B6.*G1*^*1.05*^ survived for 10 months (Fig. 2D), aligning closely with the outcomes observed in C3.*G1*^*1.05*^, C.*G1*^*1.05*^, and D2.*G1*^*1.05*^ (Fig. 1A). When analyzing N2-B6.*G1*^*1.05*^ and N2-C.*G1*^*1.0*5^ that were generated by crossbreeding C:B6.*G1*^*1.05*^ with C57BL/6J and BALB/c inbred males, respectively, we observed that N2-B6.*G1*^*1.05*^, but not N2-C.*G1*^*1.05*^, had a notably earlier mortality than F1-C:B6.*G1*^*1.05*^ (Fig. 2D). Meanwhile, N2-B6.*G1*^*1.05*^ showed a significantly higher survival rate compared with B6.*G*^*1.05*^ used in the discovery cohort study (Supplementary Fig. 1B). We postulate that the earlier mortality observed in N2-B6.*G1*^*1.05*^ is attributable to the genetic characteristics inherent in loci being homozygous for C57BL/6J inbred strain. The mortality rates in these cohort studies showed strong correlations with leukemogenesis (Fig. 2E,F). Taken together, it appears that the 129X1/SvJ strain possesses autosomal dominant traits while the C57BL/6J strain exhibits autosomal recessive traits, both of which seem to expedite the development of *Gata1.05*-driven leukemia.

The next aspect we investigated was whether strain variations influence leukemogenesis. We determined that the expression levels of GATA1 in the bone-marrow cells of wild-type female mice across BALB/c, C3H/He, and DBA/2 (with low incident), C57BL/6J and 129X1/SvJ (with high incident) inbred strains were nearly equivalent. An exception was a modest increase in C3H/He mice (Supplementary Fig. 2A). *Gata1* cDNA sequences across these strains, accessible from the Ensemble Genome Browser, were identical. We found no strong correlation between typical hematopoietic indices in wild-type female mice and the incidence rate of *Gata1.05*-driven leukemia (Supplementary Fig. 2B, C). Given these findings, we conclude that there are yet unidentified genetic modifiers influencing GATA1-deficiency and its role in *Gata1.05*-driven leukemogenesis (Supplementary Fig. 3).

This study offers persuasive evidence that inherent genetic backgrounds interact with disease-causing mutations, like the *Gata1.05* mutation, to influence the progression of leukemia. Considering the working model that supports a stepwise malignant transformation during carcinogenesis, we surmise that genetic variations relevant to the oncogenic process may give rise to synergistic or antagonistic effects on the clinical phenotype caused by primary oncogenic mutations. Our findings illuminate that among the group of polymorphisms typically considered silent, there are variations that can modify susceptibility to disease onset when influenced by genetic mutations associated with leukemia.

### Electronic supplementary material

Below is the link to the electronic supplementary material.

Supplementary Material 1

## Data Availability

All data generated or analyzed during this study are included in this published article and its supplementary information file.

## References

[1] Yamamoto M, Ko LJ, Leonard MW, Beug H, Orkin SH, Engel JD (1990). Activity and tissue-specific expression of the transcription factor NF-E1 multigene family. Genes Dev.

[2] Hirasawa R, Shimizu R, Takahashi S (2002). Essential and instructive roles of GATA factors in eosinophil development. J Exp Med.

[3] Migliaccio AR, Rana RA, Sanchez M (2003). GATA-1 as a regulator of mast cell differentiation revealed by the phenotype of the GATA-1low mouse mutant. J Exp Med.

[4] Shimizu R, Yamamoto M (2016). GATA-related hematologic disorders. Exp Hematol.

[5] Takahashi S, Onodera K, Motohashi H (1997). Arrest in primitive erythroid cell development caused by promoter-specific disruption of the GATA-1 gene. J Biol Chem.

[6] Shimizu R, Kuroha T, Ohneda O (2004). Leukemogenesis caused by incapacitated GATA-1 function. Mol Cell Biol.

[7] Pan X, Ohneda O, Ohneda K (2005). Graded levels of GATA-1 expression modulate survival, proliferation, and differentiation of erythroid progenitors. J Biol Chem.

[8] Shimizu R, Engel JD, Yamamoto M (2008). GATA1-related leukaemias. Nat Rev Cancer.

[9] Abe K, Shimizu R, Pan X, Hamada H, Yoshikawa H, Yamamoto M (2009). Stem cells of GATA1-related leukemia undergo pernicious changes after 5-fluorouracil treatment. Exp Hematol.

[10] Wang W, Wang SA, Medeiros LJ, Khoury JD (2017). Pure erythroid leukemia. Am J Hematol.

