# Supplementary material for: Strain-dependent modifiers exacerbate familial leukemia caused by GATA1-deficiency
Source: Exp Hematol Oncol. 2024 Feb 26;13:23. doi: 10.1186/s40164-024-00491-w (PMC10895851; doi:10.1186/s40164-024-00491-w)
Supplement: Supplementary file 1 — Supplementary Material 1 [file 40164_2024_491_MOESM1_ESM.pdf]

Strain-dependent modifiers exacerbate familial leukemia caused by GATA1-deficiency

Ikuo Hirano, Kanako Abe, James Douglas Engel, Masayuki Yamamoto, and Ritsuko Shimizu

List of Supporting Information

Supplementary Fig. 1: Changes in the survival rate of 129.*G1*$^{1.05}$ and *B6.G1*$^{1.05}$ based on the increase of 129X1/SvJ and C57BL/6J backgrounds through multiple crossings.

Supplementary Fig. 2: Strain differences of wild-type inbred mice in hematopoiesis.

Supplementary Fig. 3: A schematic diagram illustrating the modifiers involved in *Gata1.05*-driven leukemogenesis.

Supplementary Table 1: List of mice used for experiment shown in Figure 1

Supplementary Table 2: List of mice used for experiment shown in Figure 2C, E

Supplementary Table 3: List of mice used for experiment shown in Figure 2D, F

Supplementary information: Materials and Methods

A

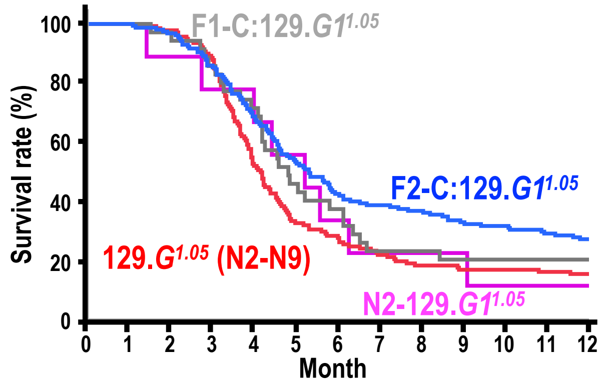

	129.*G*[1.05] (N2-N9)
F1-C:129.*G1*[1.05]	0.2831<br>0.2276
N2-129.*G1*[1.05]	0.7481<br>0.5711
F2-C:129.*G1*[1.05]	0.0015<br>0.0030

Upper row: log-rank test
Lower row: generalized Wilcoxon test

B

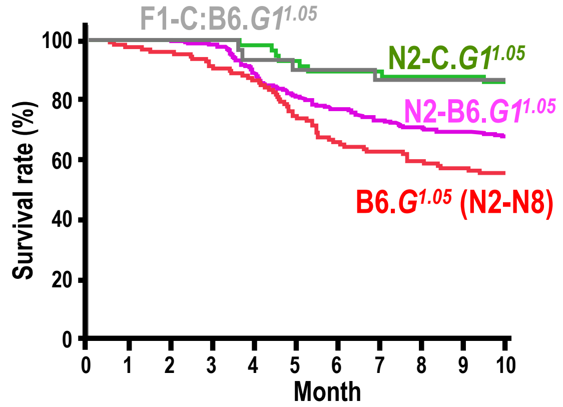

	B6.*G*[1.05] (N2-N8)
F1-C:B6.*G1*[1.05]	0.0034<br>0.0043
N2-C.*G1*[1.05]	<0.0001<br><0.0001
N2-B6.*G1*[1.05]	0.0173<br>0.0193

Upper row: log-rank test
Lower row: generalized Wilcoxon test

Supplementary Fig. 1 Changes in the survival rate of 129.*G1*$^{1.05}$ and *B6.G1*$^{1.05}$ based on the increase of 129X1/SvJ and C57BL/6J backgrounds through multiple crossings.

Comparisons of survival rates of 129.*G1*$^{1.05}$ (red line in (A)) and *B6.G1*$^{1.05}$ (red line in (B)) to those once or twice crossings as indicted. P-values resulting from the log-rank test and generalized Wilcoxon test are presented in the right panels. Autosomes in N2-129.*G1*$^{1.05}$ (pink line) are either homozygous for 129X1/SvJ or heterozygous for 129X1/SvJ and BALB/c, whereas those in F1-C:129.*G1*$^{1.05}$ (gray line) are exclusively heterozygous for 129X1/SvJ and BALB/c. F2-C:129.*G1*$^{1.05}$ (blue line) encompasses a range of backgrounds, including a homozygous 129X1/SvJ background, a heterozygous 129X1/SvJ and BALB/c backgrounds, or a homozygous BALB/c background. In contrast, autosomes in N2-B6.*G1*$^{1.05}$ (pink line) are either homozygous for C57BL/6J or heterozygous for C57BL/6J and BALB/c, and those in F1-C:B6.*G1*$^{1.05}$ (gray line) are solely heterozygous for C57BL/6J and BALB/c. N2-C.*G1*$^{1.05}$ (green line) harbor a heterozygous C57BL/6J and BALB/c background, but lacks homozygous C57BL/6J autosomes. Notably, the early mortality of 129.*G1*$^{1.05}$ remains unaffected by whether the background is heterozygosity or homozygosity for 129X1/SvJ (red vs. gray lines in (A)), whereas the early mortality of *B6.G1*$^{1.05}$ is restored in the context of C57BL/6J:BALB/c heterozygosity (red vs. gray lines in (B)). The mortality of *G1*$^{1.05}$ in a C57BL/6J background increases due to the background relationship with C57BL/6J (red vs. pink lines in (B)).

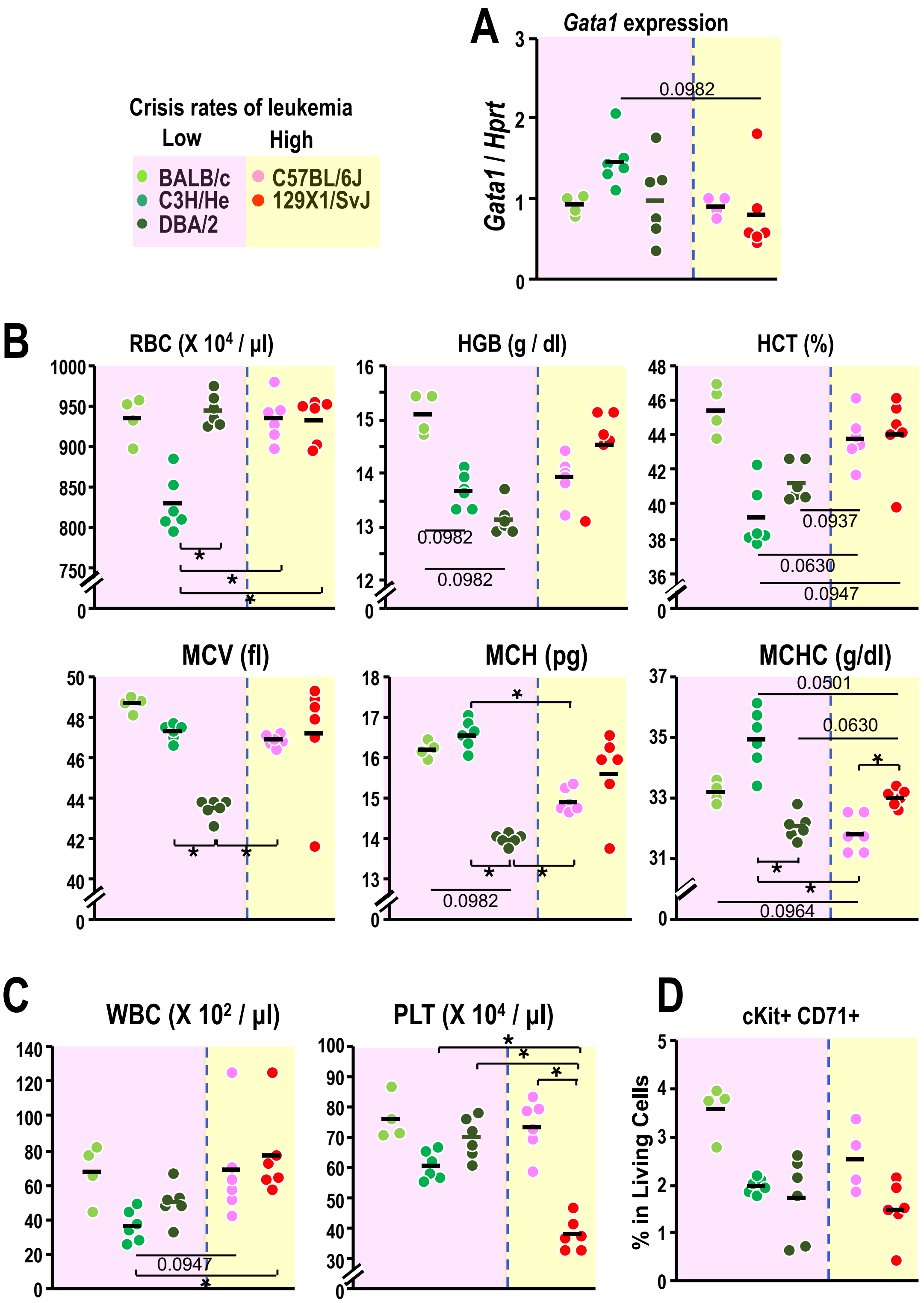
Crisis rates of leukemia
Low
High
BALB/c
C3H/He
DBA/2
C57BL/6J
129X1/SvJ
A
Gata1 expression
Gata1 / Hprt
0.0982
B
RBC (X 10^4 / µl)
HGB (g / dl)
0.0982
0.0982
HCT (%)
0.0937
0.0630
0.0947
MCV (fl)
MCH (pg)
0.0982
MCHC (g/dl)
0.0501
0.0630
0.0964
C
WBC (X 10^2 / µl)
0.0947
PLT (X 10^4 / µl)
D
cKit+ CD71+
% in Living Cells

Supplementary Fig. 2 Strain differences of wild-type inbred mice in hematopoiesis. **A** Dot plots of *Gata1* mRNA expression in the bone marrow relative to the *Hprt* housekeeping gene. **B, C** Dot plots of the indicated values for erythroid parameters (**B**) and white blood cell and platelet counts (**C**). **D** Dot plots of bone marrow erythroid populations carrying immunophenotype cKit(+)CD71(+). Results obtained from 4 BALB/c, 6 C3H, 6 DBA/2, 6 C57BL/6J and 6 129X1/SvJ wild-type female mice are depicted. Data of strains with low and high incidence of *Gata1.05* mutation-driven leukemia are discriminated by pink and yellow backgrounds, respectively. *; <0.05.

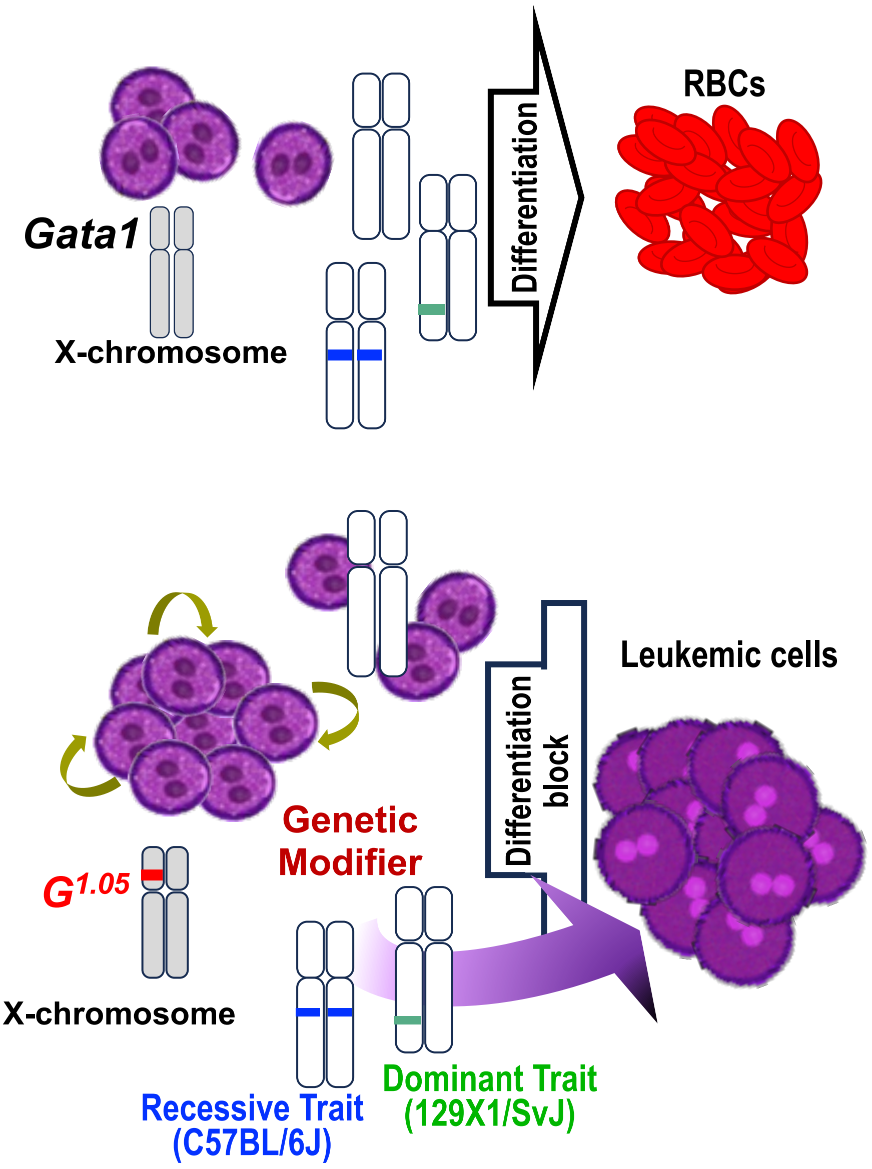

Supplementary Fig. 3 A schematic diagram illustrating the modifiers involved in *Gata1.05*-driven leukemogenesis.

Erythroid progenitors with wild-type GATA1 can differentiate normally into mature red blood cells regardless of the genetic background of mouse strains (upper panel). In contrast, erythroid progenitors harboring the *Gata1.05* mutation lack the ability to differentiate normally and remain in an immature stage that is susceptible to transforming into leukemic cells. The risk of transformation for these immature erythroid progenitors

is significantly influenced by the genetic backgrounds in terms of dominant or recessive traits (lower panel).

Supplementary Table S1. List of mice used for experiment shown in Figure 1.

*: Mice that underwent necropsy

No.		ID	Age (day)	Background	Outcome
1	*	078	120	129.*G1*$^{1.05}$(N2)	Leukemia
2	*	148	161	129.*G1*$^{1.05}$(N2)	Leukemia
3	*	151	137	129.*G1*$^{1.05}$(N2)	Leukemia
4	*	184	211	129.*G1*$^{1.05}$(N2)	Leukemia
5	*	185	124	129.*G1*$^{1.05}$(N2)	Leukemia
6	*	190	238	129.*G1*$^{1.05}$(N2)	Leukemia
7	*	196	133	129.*G1*$^{1.05}$(N2)	Leukemia
8	*	258	137	129.*G1*$^{1.05}$(N2)	Leukemia
9	*	261	100	129.*G1*$^{1.05}$(N2)	Leukemia
10	*	262	107	129.*G1*$^{1.05}$(N2)	Leukemia
11	*	298	182	129.*G1*$^{1.05}$(N2)	Leukemia
12	*	299	127	129.*G1*$^{1.05}$(N2)	Leukemia
13	*	302	128	129.*G1*$^{1.05}$(N2)	Leukemia
14	*	309	100	129.*G1*$^{1.05}$(N2)	Leukemia
15	*	337	151	129.*G1*$^{1.05}$(N2)	Leukemia
16	*	338	66	129.*G1*$^{1.05}$(N2)	Leukemia
17	*	345	147	129.*G1*$^{1.05}$(N2)	Leukemia
18	*	375	183	129.*G1*$^{1.05}$(N2)	Leukemia
19	*	376	127	129.*G1*$^{1.05}$(N2)	Leukemia
20	*	385	91	129.*G1*$^{1.05}$(N2)	Leukemia
21	*	420	128	129.*G1*$^{1.05}$(N2)	Leukemia
22	*	423	223	129.*G1*$^{1.05}$(N2)	Leukemia
23	*	457	92	129.*G1*$^{1.05}$(N2)	Leukemia
24	*	461	112	129.*G1*$^{1.05}$(N2)	Leukemia
25	*	513	73	129.*G1*$^{1.05}$(N2)	Leukemia
26		079	173	129.*G1*$^{1.05}$(N2)	Unknown
27		183	111	129.*G1*$^{1.05}$(N2)	Unknown
28		187	111	129.*G1*$^{1.05}$(N2)	Unknown
29		195	107	129.*G1*$^{1.05}$(N2)	Unknown
30		259	174	129.*G1*$^{1.05}$(N2)	Unknown
31		270	92	129.*G1*$^{1.05}$(N2)	Unknown
32		379	183	129.*G1*$^{1.05}$(N2)	Unknown
33		421	119	129.*G1*$^{1.05}$(N2)	Unknown
34		428	110	129.*G1*$^{1.05}$(N2)	Unknown
35		449	106	129.*G1*$^{1.05}$(N2)	Unknown
36	*	260	96	129.*G1*$^{1.05}$(N2)	Unknown
37		077	365	129.*G1*$^{1.05}$(N2)	Surviving
38		144	365	129.*G1*$^{1.05}$(N2)	Surviving
39		191	365	129.*G1*$^{1.05}$(N2)	Surviving
40		192	365	129.*G1*$^{1.05}$(N2)	Surviving

Supplementary Table S1 (Continued)

No.		ID	Age (day)	Background	Outcome
41	*	013	123	129.$G1^{1.05}$(N3)	Leukemia
42	*	049	53	129.$G1^{1.05}$(N3)	Leukemia
43	*	050	117	129.$G1^{1.05}$(N3)	Leukemia
44	*	150	269	129.$G1^{1.05}$(N3)	Leukemia
45	*	151	170	129.$G1^{1.05}$(N3)	Leukemia
46	*	152	101	129.$G1^{1.05}$(N3)	Leukemia
47	*	334	232	129.$G1^{1.05}$(N3)	Leukemia
48	*	336	97	129.$G1^{1.05}$(N3)	Leukemia
49	*	417	126	129.$G1^{1.05}$(N3)	Leukemia
50	*	418	161	129.$G1^{1.05}$(N3)	Leukemia
51	*	423	99	129.$G1^{1.05}$(N3)	Leukemia
52	*	445	111	129.$G1^{1.05}$(N3)	Leukemia
53	*	447	210	129.$G1^{1.05}$(N3)	Leukemia
54	*	448	121	129.$G1^{1.05}$(N3)	Leukemia
55	*	472	44	129.$G1^{1.05}$(N3)	Leukemia
56	*	474	85	129.$G1^{1.05}$(N3)	Leukemia
57	*	475	119	129.$G1^{1.05}$(N3)	Leukemia
58	*	480	101	129.$G1^{1.05}$(N3)	Leukemia
59	*	536	65	129.$G1^{1.05}$(N3)	Leukemia
60	*	562	117	129.$G1^{1.05}$(N3)	Leukemia
61	*	563	99	129.$G1^{1.05}$(N3)	Leukemia
62	*	565	111	129.$G1^{1.05}$(N3)	Leukemia
63	*	569	86	129.$G1^{1.05}$(N3)	Leukemia
64	*	572	119	129.$G1^{1.05}$(N3)	Leukemia
65	*	614	92	129.$G1^{1.05}$(N3)	Leukemia
66	*	665	140	129.$G1^{1.05}$(N3)	Leukemia
67	*	667	120	129.$G1^{1.05}$(N3)	Leukemia
68	*	725	101	129.$G1^{1.05}$(N3)	Leukemia
69	*	795	127	129.$G1^{1.05}$(N3)	Leukemia
70	*	838	139	129.$G1^{1.05}$(N3)	Leukemia
71	*	843	120	129.$G1^{1.05}$(N3)	Leukemia
72		116	328	129.$G1^{1.05}$(N3)	Unknown
73		422	351	129.$G1^{1.05}$(N3)	Unknown
74		459	206	129.$G1^{1.05}$(N3)	Unknown
75		573	119	129.$G1^{1.05}$(N3)	Unknown
76		612	148	129.$G1^{1.05}$(N3)	Unknown
77		672	270	129.$G1^{1.05}$(N3)	Unknown
78	*	545	82	129.$G1^{1.05}$(N3)	Unknown
79		117	365	129.$G1^{1.05}$(N3)	Surviving
80		333	365	129.$G1^{1.05}$(N3)	Surviving

Supplementary Table S1 (Continued)

No.		ID	Age (day)	Background	Outcome
81		666	365	129.$G1^{1.05}$(N3)	Surviving
82		667	365	129.$G1^{1.05}$(N3)	Surviving
83	*	120	116	129.$G1^{1.05}$(N4)	Leukemia
84	*	611	96	129.$G1^{1.05}$(N4)	Leukemia
85	*	615	144	129.$G1^{1.05}$(N4)	Leukemia
86	*	632	141	129.$G1^{1.05}$(N4)	Leukemia
87	*	674	224	129.$G1^{1.05}$(N4)	Leukemia
88	*	890	72	129.$G1^{1.05}$(N4)	Leukemia
89		613	115	129.$G1^{1.05}$(N4)	Unknown
90		614	157	129.$G1^{1.05}$(N4)	Unknown
91		616	66	129.$G1^{1.05}$(N4)	Unknown
92		673	77	129.$G1^{1.05}$(N4)	Unknown
93		675	96	129.$G1^{1.05}$(N4)	Unknown
94		676	132	129.$G1^{1.05}$(N4)	Unknown
95		891	104	129.$G1^{1.05}$(N4)	Unknown
96		521	365	129.$G1^{1.05}$(N4)	Surviving
97		595	365	129.$G1^{1.05}$(N4)	Surviving
98		603	365	129.$G1^{1.05}$(N4)	Surviving
99	*	032	94	129.$G1^{1.05}$(N5)	Leukemia
100	*	046	129	129.$G1^{1.05}$(N5)	Leukemia
101	*	641	128	129.$G1^{1.05}$(N5)	Leukemia
102	*	698	186	129.$G1^{1.05}$(N5)	Leukemia
103	*	813	109	129.$G1^{1.05}$(N5)	Leukemia
104	*	814	97	129.$G1^{1.05}$(N5)	Leukemia
105		039	99	129.$G1^{1.05}$(N5)	Unknown
106		045	116	129.$G1^{1.05}$(N5)	Unknown
107		162	106	129.$G1^{1.05}$(N5)	Unknown
108		811	94	129.$G1^{1.05}$(N5)	Unknown
109		044	365	129.$G1^{1.05}$(N5)	Surviving
110		096	365	129.$G1^{1.05}$(N5)	Surviving
111		419	365	129.$G1^{1.05}$(N5)	Surviving
112		696	365	129.$G1^{1.05}$(N5)	Surviving
113		697	365	129.$G1^{1.05}$(N5)	Surviving
114		699	365	129.$G1^{1.05}$(N5)	Surviving
115		816	365	129.$G1^{1.05}$(N5)	Surviving
116	*	087	218	129.$G1^{1.05}$(N6)	Leukemia
117	*	149	147	129.$G1^{1.05}$(N6)	Leukemia
118	*	198	143	129.$G1^{1.05}$(N6)	Leukemia
119	*	206	133	129.$G1^{1.05}$(N6)	Leukemia
120	*	318	114	129.$G1^{1.05}$(N6)	Leukemia

Supplementary Table S1 (Continued)

No.		ID	Age (day)	Background	Outcome
121	*	319	90	129.*G1*$^{1.05}$(N6)	Leukemia
122	*	440	106	129.*G1*$^{1.05}$(N6)	Leukemia
123	*	742	102	129.*G1*$^{1.05}$(N6)	Leukemia
124	*	801	88	129.*G1*$^{1.05}$(N6)	Leukemia
125		048	93	129.*G1*$^{1.05}$(N6)	Unknown
126		219	92	129.*G1*$^{1.05}$(N6)	Unknown
127		244	105	129.*G1*$^{1.05}$(N6)	Unknown
128		488	190	129.*G1*$^{1.05}$(N6)	Unknown
129		074	365	129.*G1*$^{1.05}$(N6)	Surviving
130		086	365	129.*G1*$^{1.05}$(N6)	Surviving
131		146	365	129.*G1*$^{1.05}$(N6)	Surviving
132	*	390	145	129.*G1*$^{1.05}$(N7)	Leukemia
133		339	103	129.*G1*$^{1.05}$(N7)	Unknown
134		340	198	129.*G1*$^{1.05}$(N7)	Unknown
135		341	50	129.*G1*$^{1.05}$(N7)	Unknown
136		391	146	129.*G1*$^{1.05}$(N7)	Unknown
137	*	603	84	129.*G1*$^{1.05}$(N8)	Leukemia
138		757	138	129.*G1*$^{1.05}$(N9)	Unknown
139		758	83	129.*G1*$^{1.05}$(N9)	Unknown
140	*	368	157	B6.*G1*$^{1.05}$(N2)	Leukemia
141	*	580	123	B6.*G1*$^{1.05}$(N2)	Leukemia
142	*	675	161	B6.*G1*$^{1.05}$(N2)	Leukemia
143		226	27	B6.*G1*$^{1.05}$(N2)	Unknown
144		249	144	B6.*G1*$^{1.05}$(N2)	Unknown
145		253	164	B6.*G1*$^{1.05}$(N2)	Unknown
146		255	164	B6.*G1*$^{1.05}$(N2)	Unknown
147		258	90	B6.*G1*$^{1.05}$(N2)	Unknown
148		259	364	B6.*G1*$^{1.05}$(N2)	Unknown
149		267	147	B6.*G1*$^{1.05}$(N2)	Unknown
150		365	87	B6.*G1*$^{1.05}$(N2)	Unknown
151		369	87	B6.*G1*$^{1.05}$(N2)	Unknown
152		539	137	B6.*G1*$^{1.05}$(N2)	Unknown
153		644	124	B6.*G1*$^{1.05}$(N2)	Unknown
154		646	19	B6.*G1*$^{1.05}$(N2)	Unknown
155		743	75	B6.*G1*$^{1.05}$(N2)	Unknown
156		748	39	B6.*G1*$^{1.05}$(N2)	Unknown
157		828	164	B6.*G1*$^{1.05}$(N2)	Unknown
158		873	144	B6.*G1*$^{1.05}$(N2)	Unknown
159		C710	150	B6.*G1*$^{1.05}$(N2)	Unknown
160	*	644	226	B6.*G1*$^{1.05}$(N2)	Unknown

Supplementary Table S1 (Continued)

No.		ID	Age (day)	Background	Outcome
161	*	775	315	B6.$G1^{1.05}$(N2)	Unknown
162		140	365	B6.$G1^{1.05}$(N2)	Surviving
163		142	365	B6.$G1^{1.05}$(N2)	Surviving
164		160	365	B6.$G1^{1.05}$(N2)	Surviving
165		161	365	B6.$G1^{1.05}$(N2)	Surviving
166		165	365	B6.$G1^{1.05}$(N2)	Surviving
167		188	365	B6.$G1^{1.05}$(N2)	Surviving
168		230	365	B6.$G1^{1.05}$(N2)	Surviving
169		255	365	B6.$G1^{1.05}$(N2)	Surviving
170		383	365	B6.$G1^{1.05}$(N2)	Surviving
171		670	365	B6.$G1^{1.05}$(N2)	Surviving
172		764	365	B6.$G1^{1.05}$(N2)	Surviving
173	*	020	141	B6.$G1^{1.05}$(N3)	Leukemia
174	*	218	130	B6.$G1^{1.05}$(N3)	Leukemia
175	*	237	118	B6.$G1^{1.05}$(N3)	Leukemia
176	*	329	181	B6.$G1^{1.05}$(N3)	Leukemia
177	*	363	184	B6.$G1^{1.05}$(N3)	Leukemia
178	*	430	176	B6.$G1^{1.05}$(N3)	Leukemia
179	*	447	253	B6.$G1^{1.05}$(N3)	Leukemia
180	*	543	273	B6.$G1^{1.05}$(N3)	Leukemia
181	*	553	143	B6.$G1^{1.05}$(N3)	Leukemia
182	*	566	241	B6.$G1^{1.05}$(N3)	Leukemia
183	*	567	229	B6.$G1^{1.05}$(N3)	Leukemia
184	*	681	173	B6.$G1^{1.05}$(N3)	Leukemia
185	*	739	85	B6.$G1^{1.05}$(N3)	Leukemia
186	*	748	315	B6.$G1^{1.05}$(N3)	Leukemia
187	*	791	165	B6.$G1^{1.05}$(N3)	Leukemia
188		019	62	B6.$G1^{1.05}$(N3)	Unknown
189		401	229	B6.$G1^{1.05}$(N3)	Unknown
190		407	111	B6.$G1^{1.05}$(N3)	Unknown
191		457	138	B6.$G1^{1.05}$(N3)	Unknown
192		495	133	B6.$G1^{1.05}$(N3)	Unknown
193		602	136	B6.$G1^{1.05}$(N3)	Unknown
194		789	147	B6.$G1^{1.05}$(N3)	Unknown
195		877	103	B6.$G1^{1.05}$(N3)	Unknown
196		017	365	B6.$G1^{1.05}$(N3)	Surviving
197		043	365	B6.$G1^{1.05}$(N3)	Surviving
198		064	365	B6.$G1^{1.05}$(N3)	Surviving
199		080	365	B6.$G1^{1.05}$(N3)	Surviving
200		081	365	B6.$G1^{1.05}$(N3)	Surviving

Supplementary Table S1 (Continued)

No.		ID	Age (day)	Background	Outcome
201		238	365	B6.$G1^{1.05}$(N3)	Surviving
202		239	365	B6.$G1^{1.05}$(N3)	Surviving
203		335	365	B6.$G1^{1.05}$(N3)	Surviving
204		444	365	B6.$G1^{1.05}$(N3)	Surviving
205		541	365	B6.$G1^{1.05}$(N3)	Surviving
206		552	365	B6.$G1^{1.05}$(N3)	Surviving
207		555	365	B6.$G1^{1.05}$(N3)	Surviving
208		561	365	B6.$G1^{1.05}$(N3)	Surviving
209		568	365	B6.$G1^{1.05}$(N3)	Surviving
210		595	365	B6.$G1^{1.05}$(N3)	Surviving
211		596	365	B6.$G1^{1.05}$(N3)	Surviving
212		610	365	B6.$G1^{1.05}$(N3)	Surviving
213		668	365	B6.$G1^{1.05}$(N3)	Surviving
214		670	365	B6.$G1^{1.05}$(N3)	Surviving
215		677	365	B6.$G1^{1.05}$(N3)	Surviving
216		740	365	B6.$G1^{1.05}$(N3)	Surviving
217		743	365	B6.$G1^{1.05}$(N3)	Surviving
218		747	365	B6.$G1^{1.05}$(N3)	Surviving
219		750	365	B6.$G1^{1.05}$(N3)	Surviving
220		788	365	B6.$G1^{1.05}$(N3)	Surviving
221		789	365	B6.$G1^{1.05}$(N3)	Surviving
222		814	365	B6.$G1^{1.05}$(N3)	Surviving
223		817	365	B6.$G1^{1.05}$(N3)	Surviving
224		848	365	B6.$G1^{1.05}$(N3)	Surviving
225		849	365	B6.$G1^{1.05}$(N3)	Surviving
226	*	236	281	B6.$G1^{1.05}$(N4)	Leukemia
227	*	263	127	B6.$G1^{1.05}$(N4)	Leukemia
228	*	322	251	B6.$G1^{1.05}$(N4)	Leukemia
229	*	514	328	B6.$G1^{1.05}$(N4)	Leukemia
230	*	865	229	B6.$G1^{1.05}$(N4)	Leukemia
231		275	135	B6.$G1^{1.05}$(N4)	Unknown
232		277	338	B6.$G1^{1.05}$(N4)	Unknown
233		744	165	B6.$G1^{1.05}$(N4)	Unknown
234		124	365	B6.$G1^{1.05}$(N4)	Surviving
235		206	365	B6.$G1^{1.05}$(N4)	Surviving
236		264	365	B6.$G1^{1.05}$(N4)	Surviving
237		671	365	B6.$G1^{1.05}$(N4)	Surviving
238		738	365	B6.$G1^{1.05}$(N4)	Surviving
239		750	365	B6.$G1^{1.05}$(N4)	Surviving
240		781	365	B6.$G1^{1.05}$(N4)	Surviving

Supplementary Table S1 (Continued)

No.		ID	Age (day)	Background	Outcome
241		793	365	B6.$G1^{1.05}$(N4)	Surviving
242		824	365	B6.$G1^{1.05}$(N4)	Surviving
243		825	365	B6.$G1^{1.05}$(N4)	Surviving
244		839	365	B6.$G1^{1.05}$(N4)	Surviving
245		881	365	B6.$G1^{1.05}$(N4)	Surviving
246	*	891	200	B6.$G1^{1.05}$(N5)	Leukemia
247		199	45	B6.$G1^{1.05}$(N5)	Unknown
248		620	16	B6.$G1^{1.05}$(N5)	Unknown
249		893	198	B6.$G1^{1.05}$(N5)	Unknown
250		058	365	B6.$G1^{1.05}$(N5)	Surviving
251		078	365	B6.$G1^{1.05}$(N5)	Surviving
252		094	365	B6.$G1^{1.05}$(N5)	Surviving
253		197	365	B6.$G1^{1.05}$(N5)	Surviving
254		217	365	B6.$G1^{1.05}$(N5)	Surviving
255		266	365	B6.$G1^{1.05}$(N5)	Surviving
256		450	365	B6.$G1^{1.05}$(N5)	Surviving
257	*	154	161	B6.$G1^{1.05}$(N6)	Leukemia
258		099	102	B6.$G1^{1.05}$(N6)	Unknown
259		100	365	B6.$G1^{1.05}$(N6)	Surviving
260		224	365	B6.$G1^{1.05}$(N6)	Surviving
261		412	365	B6.$G1^{1.05}$(N6)	Surviving
262		413	365	B6.$G1^{1.05}$(N6)	Surviving
263		468	365	B6.$G1^{1.05}$(N6)	Surviving
264		154	74	B6.$G1^{1.05}$(N8)	Unknown
265		447	115	B6.$G1^{1.05}$(N8)	Unknown
266	*	538	136	C.$G1^{1.05}$(N2)	Leukemia
267		605	312	C.$G1^{1.05}$(N2)	Unknown
268	*	541	85	C.$G1^{1.05}$(N2)	Unknown
269		542	365	C.$G1^{1.05}$(N2)	Surviving
270		606	365	C.$G1^{1.05}$(N2)	Surviving
271		466	175	C.$G1^{1.05}$(N3)	Unknown
272		715	142	C.$G1^{1.05}$(N3)	Unknown
273		719	264	C.$G1^{1.05}$(N3)	Unknown
274		703	365	C.$G1^{1.05}$(N3)	Surviving
275		722	365	C.$G1^{1.05}$(N3)	Surviving
276		006	318	C.$G1^{1.05}$(N4)	Unknown
277		880	201	C.$G1^{1.05}$(N4)	Unknown
278	*	035	205	C.$G1^{1.05}$(N4)	Unknown
279	*	899	165	C.$G1^{1.05}$(N4)	Unknown
280		030	365	C.$G1^{1.05}$(N4)	Surviving

Supplementary Table S1 (Continued)

No.		ID	Age (day)	Background	Outcome
281		887	365	C.*G1*$^{1.05}$(N4)	Surviving
282		115	365	C.*G1*$^{1.05}$(N5)	Surviving
283		118	365	C.*G1*$^{1.05}$(N5)	Surviving
284		122	365	C.*G1*$^{1.05}$(N5)	Surviving
285		124	365	C.*G1*$^{1.05}$(N5)	Surviving
286		169	365	C.*G1*$^{1.05}$(N5)	Surviving
287		208	292	C.*G1*$^{1.05}$(N6)	Unknown
288		210	365	C.*G1*$^{1.05}$(N6)	Surviving
289		214	365	C.*G1*$^{1.05}$(N6)	Surviving
290		256	365	C.*G1*$^{1.05}$(N6)	Surviving
291		320	365	C.*G1*$^{1.05}$(N6)	Surviving
292		321	365	C.*G1*$^{1.05}$(N6)	Surviving
293		362	365	C.*G1*$^{1.05}$(N6)	Surviving
294		367	365	C.*G1*$^{1.05}$(N6)	Surviving
295		367	365	C.*G1*$^{1.05}$(N6)	Surviving
296		486	365	C.*G1*$^{1.05}$(N6)	Surviving
297		088	365	C.*G1*$^{1.05}$(N7)	Surviving
298		248	365	C.*G1*$^{1.05}$(N7)	Surviving
299		329	365	C.*G1*$^{1.05}$(N7)	Surviving
300		447	365	C.*G1*$^{1.05}$(N8)	Surviving
301		502	365	C.*G1*$^{1.05}$(N8)	Surviving
302		540	365	C.*G1*$^{1.05}$(N8)	Surviving
303		541	365	C.*G1*$^{1.05}$(N8)	Surviving
304		544	365	C.*G1*$^{1.05}$(N8)	Surviving
305		545	365	C.*G1*$^{1.05}$(N8)	Surviving
306		546	365	C.*G1*$^{1.05}$(N8)	Surviving
307		588	365	C.*G1*$^{1.05}$(N9)	Surviving
308		618	365	C.*G1*$^{1.05}$(N9)	Surviving
309		857	365	C.*G1*$^{1.05}$(N9)	Surviving
310		255	365	C3.*G1*$^{1.05}$(N2)	Surviving
311		257	365	C3.*G1*$^{1.05}$(N2)	Surviving
312		314	365	C3.*G1*$^{1.05}$(N2)	Surviving
313		407	365	C3.*G1*$^{1.05}$(N2)	Surviving
314		408	365	C3.*G1*$^{1.05}$(N2)	Surviving
315	*	431	145	C3.*G1*$^{1.05}$(N3)	Leukemia
316	*	528	273	C3.*G1*$^{1.05}$(N3)	Unknown
317		426	365	C3.*G1*$^{1.05}$(N3)	Surviving
318		485	365	C3.*G1*$^{1.05}$(N3)	Surviving
319		486	365	C3.*G1*$^{1.05}$(N3)	Surviving
320		525	365	C3.*G1*$^{1.05}$(N3)	Surviving

Supplementary Table S1 (Continued)

No.		ID	Age (day)	Background	Outcome
321		526	365	C3.$G1^{1.05}$(N3)	Surviving
322		768	365	C3.$G1^{1.05}$(N4)	Surviving
323		801	365	C3.$G1^{1.05}$(N4)	Surviving
324		854	365	C3.$G1^{1.05}$(N4)	Surviving
325	*	402	66	C3.$G1^{1.05}$(N5)	Unknown
326		224	365	C3.$G1^{1.05}$(N5)	Surviving
327		401	365	C3.$G1^{1.05}$(N5)	Surviving
328		417	365	C3.$G1^{1.05}$(N5)	Surviving
329		011	365	C3.$G1^{1.05}$(N6)	Surviving
330		200	14	D2.$G1^{1.05}$(N2)	Unknown
331		599	124	D2.$G1^{1.05}$(N2)	Unknown
332		613	266	D2.$G1^{1.05}$(N2)	Unknown
333	*	267	235	D2.$G1^{1.05}$(N2)	Unknown
334		158	365	D2.$G1^{1.05}$(N2)	Surviving
335		222	365	D2.$G1^{1.05}$(N2)	Surviving
336		223	365	D2.$G1^{1.05}$(N2)	Surviving
337		224	365	D2.$G1^{1.05}$(N2)	Surviving
338		264	365	D2.$G1^{1.05}$(N2)	Surviving
339		265	365	D2.$G1^{1.05}$(N2)	Surviving
340		532	365	D2.$G1^{1.05}$(N2)	Surviving
341		610	365	D2.$G1^{1.05}$(N2)	Surviving
342		611	365	D2.$G1^{1.05}$(N2)	Surviving
343		614	365	D2.$G1^{1.05}$(N2)	Surviving
344		615	365	D2.$G1^{1.05}$(N2)	Surviving
345		563	297	D2.$G1^{1.05}$(N3)	Unknown
346		779	114	D2.$G1^{1.05}$(N3)	Unknown
347		492	365	D2.$G1^{1.05}$(N3)	Surviving
348		505	365	D2.$G1^{1.05}$(N3)	Surviving
349		507	365	D2.$G1^{1.05}$(N3)	Surviving
350		510	365	D2.$G1^{1.05}$(N3)	Surviving
351		564	365	D2.$G1^{1.05}$(N3)	Surviving
352		595	365	D2.$G1^{1.05}$(N3)	Surviving
353		658	365	D2.$G1^{1.05}$(N3)	Surviving
354		677	365	D2.$G1^{1.05}$(N3)	Surviving
355		694	365	D2.$G1^{1.05}$(N3)	Surviving
356		736	365	D2.$G1^{1.05}$(N3)	Surviving
357		748	365	D2.$G1^{1.05}$(N3)	Surviving
358	*	018	157	D2.$G1^{1.05}$(N4)	Unknown
359	*	019	47	D2.$G1^{1.05}$(N4)	Unknown
360	*	746	281	D2.$G1^{1.05}$(N4)	Unknown

Supplementary Table S1 (Continued)

No.		ID	Age (day)	Background	Outcome
361		566	365	D2.*G1*$^{1.05}$(N4)	Surviving
362		569	365	D2.*G1*$^{1.05}$(N4)	Surviving
363		581	365	D2.*G1*$^{1.05}$(N4)	Surviving
364		605	365	D2.*G1*$^{1.05}$(N4)	Surviving
365		744	365	D2.*G1*$^{1.05}$(N4)	Surviving
366		747	365	D2.*G1*$^{1.05}$(N4)	Surviving
367		790	365	D2.*G1*$^{1.05}$(N4)	Surviving
368		052	365	D2.*G1*$^{1.05}$(N5)	Surviving
369	*	278	49	D2.*G1*$^{1.05}$(N6)	Unknown

Supplementary Table S2. List of mice used for experiment shown in Figure 2C, E.

*: Mice that underwent necropsy

No.		ID	Age (day)	Background	Outcome
1	*	670	158	N2 129.*G1*$^{1.05}$	Leukemia
2	*	671	169	N2 129.*G1*$^{1.05}$	Leukemia
3	*	755	121	N2 129.*G1*$^{1.05}$	Leukemia
4	*	759	190	N2 129.*G1*$^{1.05}$	Leukemia
5	*	763	134	N2 129.*G1*$^{1.05}$	Leukemia
6		669	83	N2 129.*G1*$^{1.05}$	Unknown
7		752	276	N2 129.*G1*$^{1.05}$	Unknown
8		753	43	N2 129.*G1*$^{1.05}$	Unknown
9		668	365	N2 129.*G1*$^{1.05}$	Surviving
10	*	032	97	F1 C:129.*G1*$^{1.05}$	Leukemia
11	*	085	186	F1 C:129.*G1*$^{1.05}$	Leukemia
12	*	087	61	F1 C:129.*G1*$^{1.05}$	Leukemia
13	*	089	88	F1 C:129.*G1*$^{1.05}$	Leukemia
14	*	095	46	F1 C:129.*G1*$^{1.05}$	Leukemia
15	*	140	203	F1 C:129.*G1*$^{1.05}$	Leukemia
16	*	361	111	F1 C:129.*G1*$^{1.05}$	Leukemia
17	*	362	186	F1 C:129.*G1*$^{1.05}$	Leukemia
18	*	403	99	F1 C:129.*G1*$^{1.05}$	Leukemia
19	*	404	198	F1 C:129.*G1*$^{1.05}$	Leukemia
20	*	405	153	F1 C:129.*G1*$^{1.05}$	Leukemia
21	*	502	158	F1 C:129.*G1*$^{1.05}$	Leukemia
22	*	560	126	F1 C:129.*G1*$^{1.05}$	Leukemia
23	*	561	129	F1 C:129.*G1*$^{1.05}$	Leukemia
24	*	562	93	F1 C:129.*G1*$^{1.05}$	Leukemia
25	*	619	256	F1 C:129.*G1*$^{1.05}$	Leukemia
26	*	654	146	F1 C:129.*G1*$^{1.05}$	Leukemia
27	*	659	119	F1 C:129.*G1*$^{1.05}$	Leukemia
28	*	757	193	F1 C:129.*G1*$^{1.05}$	Leukemia
29	*	831	176	F1 C:129.*G1*$^{1.05}$	Leukemia
30	*	833	85	F1 C:129.*G1*$^{1.05}$	Leukemia
31		296	138	F1 C:129.*G1*$^{1.05}$	Unknown
32		359	147	F1 C:129.*G1*$^{1.05}$	Unknown
33		563	139	F1 C:129.*G1*$^{1.05}$	Unknown
34		616	127	F1 C:129.*G1*$^{1.05}$	Unknown
35		618	127	F1 C:129.*G1*$^{1.05}$	Unknown
36		660	124	F1 C:129.*G1*$^{1.05}$	Unknown
37		832	82	F1 C:129.*G1*$^{1.05}$	Unknown
38		034	365	F1 C:129.*G1*$^{1.05}$	Surviving
39		088	365	F1 C:129.*G1*$^{1.05}$	Surviving
40		148	365	F1 C:129.*G1*$^{1.05}$	Surviving

Supplementary Table S2 (Continued)

No.		ID	Age (day)	Background	Outcome
41		297	365	F1 C:129.*G1*$^{1.05}$	Surviving
42		357	365	F1 C:129.*G1*$^{1.05}$	Surviving
43		363	365	F1 C:129.*G1*$^{1.05}$	Surviving
44		501	365	F1 C:129.*G1*$^{1.05}$	Surviving
45	*	026	203	F2 C:129.*G1*$^{1.05}$	Leukemia
46	*	029	151	F2 C:129.*G1*$^{1.05}$	Leukemia
47	*	030	134	F2 C:129.*G1*$^{1.05}$	Leukemia
48	*	061	154	F2 C:129.*G1*$^{1.05}$	Leukemia
49	*	066	141	F2 C:129.*G1*$^{1.05}$	Leukemia
50	*	067	67	F2 C:129.*G1*$^{1.05}$	Leukemia
51	*	068	101	F2 C:129.*G1*$^{1.05}$	Leukemia
52	*	070	138	F2 C:129.*G1*$^{1.05}$	Leukemia
53	*	089	87	F2 C:129.*G1*$^{1.05}$	Leukemia
54	*	092	157	F2 C:129.*G1*$^{1.05}$	Leukemia
55	*	093	87	F2 C:129.*G1*$^{1.05}$	Leukemia
56	*	109	123	F2 C:129.*G1*$^{1.05}$	Leukemia
57	*	117	120	F2 C:129.*G1*$^{1.05}$	Leukemia
58	*	168	98	F2 C:129.*G1*$^{1.05}$	Leukemia
59	*	193	116	F2 C:129.*G1*$^{1.05}$	Leukemia
60	*	210	74	F2 C:129.*G1*$^{1.05}$	Leukemia
61	*	212	116	F2 C:129.*G1*$^{1.05}$	Leukemia
62	*	350	130	F2 C:129.*G1*$^{1.05}$	Leukemia
63	*	351	119	F2 C:129.*G1*$^{1.05}$	Leukemia
64	*	398	111	F2 C:129.*G1*$^{1.05}$	Leukemia
65	*	404	93	F2 C:129.*G1*$^{1.05}$	Leukemia
66	*	405	138	F2 C:129.*G1*$^{1.05}$	Leukemia
67	*	408	233	F2 C:129.*G1*$^{1.05}$	Leukemia
68	*	409	198	F2 C:129.*G1*$^{1.05}$	Leukemia
69	*	426	340	F2 C:129.*G1*$^{1.05}$	Leukemia
70	*	436	104	F2 C:129.*G1*$^{1.05}$	Leukemia
71	*	439	104	F2 C:129.*G1*$^{1.05}$	Leukemia
72	*	444	87	F2 C:129.*G1*$^{1.05}$	Leukemia
73	*	485	274	F2 C:129.*G1*$^{1.05}$	Leukemia
74	*	487	135	F2 C:129.*G1*$^{1.05}$	Leukemia
75	*	534	125	F2 C:129.*G1*$^{1.05}$	Leukemia
76	*	540	161	F2 C:129.*G1*$^{1.05}$	Leukemia
77	*	547	226	F2 C:129.*G1*$^{1.05}$	Leukemia
78	*	568	150	F2 C:129.*G1*$^{1.05}$	Leukemia
79	*	569	176	F2 C:129.*G1*$^{1.05}$	Leukemia
80	*	570	50	F2 C:129.*G1*$^{1.05}$	Leukemia

Supplementary Table S2 (Continued)

No.		ID	Age (day)	Background	Outcome
81	*	586	102	F2 C:129.$G1^{1.05}$	Leukemia
82	*	588	162	F2 C:129.$G1^{1.05}$	Leukemia
83	*	589	83	F2 C:129.$G1^{1.05}$	Leukemia
84	*	590	58	F2 C:129.$G1^{1.05}$	Leukemia
85	*	593	130	F2 C:129.$G1^{1.05}$	Leukemia
86	*	604	94	F2 C:129.$G1^{1.05}$	Leukemia
87	*	618	104	F2 C:129.$G1^{1.05}$	Leukemia
88	*	624	105	F2 C:129.$G1^{1.05}$	Leukemia
89	*	635	172	F2 C:129.$G1^{1.05}$	Leukemia
90	*	637	84	F2 C:129.$G1^{1.05}$	Leukemia
91	*	638	330	F2 C:129.$G1^{1.05}$	Leukemia
92	*	658	147	F2 C:129.$G1^{1.05}$	Leukemia
93	*	692	186	F2 C:129.$G1^{1.05}$	Leukemia
94	*	718	307	F2 C:129.$G1^{1.05}$	Leukemia
95	*	719	286	F2 C:129.$G1^{1.05}$	Leukemia
96	*	724	100	F2 C:129.$G1^{1.05}$	Leukemia
97	*	740	113	F2 C:129.$G1^{1.05}$	Leukemia
98	*	742	134	F2 C:129.$G1^{1.05}$	Leukemia
99	*	743	191	F2 C:129.$G1^{1.05}$	Leukemia
100	*	751	80	F2 C:129.$G1^{1.05}$	Leukemia
101	*	752	66	F2 C:129.$G1^{1.05}$	Leukemia
102	*	776	113	F2 C:129.$G1^{1.05}$	Leukemia
103	*	792	271	F2 C:129.$G1^{1.05}$	Leukemia
104	*	799	94	F2 C:129.$G1^{1.05}$	Leukemia
105	*	802	69	F2 C:129.$G1^{1.05}$	Leukemia
106	*	824	176	F2 C:129.$G1^{1.05}$	Leukemia
107	*	836	136	F2 C:129.$G1^{1.05}$	Leukemia
108	*	838	158	F2 C:129.$G1^{1.05}$	Leukemia
109	*	839	177	F2 C:129.$G1^{1.05}$	Leukemia
110	*	845	162	F2 C:129.$G1^{1.05}$	Leukemia
111	*	877	131	F2 C:129.$G1^{1.05}$	Leukemia
112	*	878	171	F2 C:129.$G1^{1.05}$	Leukemia
113	*	879	244	F2 C:129.$G1^{1.05}$	Leukemia
114	*	882	105	F2 C:129.$G1^{1.05}$	Leukemia
115	*	883	118	F2 C:129.$G1^{1.05}$	Leukemia
116		005	35	F2 C:129.$G1^{1.05}$	Unknown
117		010	178	F2 C:129.$G1^{1.05}$	Unknown
118		022	110	F2 C:129.$G1^{1.05}$	Unknown
119		028	86	F2 C:129.$G1^{1.05}$	Unknown
120		059	332	F2 C:129.$G1^{1.05}$	Unknown

Supplementary Table S2 (Continued)

No.	ID	Age (day)	Background	Outcome
121	129	112	F2 C:129.*G1*[1.05]	Unknown
122	130	80	F2 C:129.*G1*[1.05]	Unknown
123	157	141	F2 C:129.*G1*[1.05]	Unknown
124	160	86	F2 C:129.*G1*[1.05]	Unknown
125	203	126	F2 C:129.*G1*[1.05]	Unknown
126	220	122	F2 C:129.*G1*[1.05]	Unknown
127	224	73	F2 C:129.*G1*[1.05]	Unknown
128	250	68	F2 C:129.*G1*[1.05]	Unknown
129	326	145	F2 C:129.*G1*[1.05]	Unknown
130	329	132	F2 C:129.*G1*[1.05]	Unknown
131	353	262	F2 C:129.*G1*[1.05]	Unknown
132	354	55	F2 C:129.*G1*[1.05]	Unknown
133	394	249	F2 C:129.*G1*[1.05]	Unknown
134	401	220	F2 C:129.*G1*[1.05]	Unknown
135	402	186	F2 C:129.*G1*[1.05]	Unknown
136	403	358	F2 C:129.*G1*[1.05]	Unknown
137	528	33	F2 C:129.*G1*[1.05]	Unknown
138	534	161	F2 C:129.*G1*[1.05]	Unknown
139	538	64	F2 C:129.*G1*[1.05]	Unknown
140	595	262	F2 C:129.*G1*[1.05]	Unknown
141	645	183	F2 C:129.*G1*[1.05]	Unknown
142	645	353	F2 C:129.*G1*[1.05]	Unknown
143	648	258	F2 C:129.*G1*[1.05]	Unknown
144	666	306	F2 C:129.*G1*[1.05]	Unknown
145	723	180	F2 C:129.*G1*[1.05]	Unknown
146	726	139	F2 C:129.*G1*[1.05]	Unknown
147	749	140	F2 C:129.*G1*[1.05]	Unknown
148	756	139	F2 C:129.*G1*[1.05]	Unknown
149	772	152	F2 C:129.*G1*[1.05]	Unknown
150	775	69	F2 C:129.*G1*[1.05]	Unknown
151	778	122	F2 C:129.*G1*[1.05]	Unknown
152	796	171	F2 C:129.*G1*[1.05]	Unknown
153	799	120	F2 C:129.*G1*[1.05]	Unknown
154	834	91	F2 C:129.*G1*[1.05]	Unknown
155	837	115	F2 C:129.*G1*[1.05]	Unknown
156	842	175	F2 C:129.*G1*[1.05]	Unknown
157	880	99	F2 C:129.*G1*[1.05]	Unknown
158	* 407	94	F2 C:129.*G1*[1.05]	Unknown
159	005	365	F2 C:129.*G1*[1.05]	Surviving
160	025	365	F2 C:129.*G1*[1.05]	Surviving

Supplementary Table S2 (Continued)

No.	ID	Age (day)	Background	Outcome
161	057	365	F2 C:129.*G1*$^{1.05}$	Surviving
162	111	365	F2 C:129.*G1*$^{1.05}$	Surviving
163	120	365	F2 C:129.*G1*$^{1.05}$	Surviving
164	127	365	F2 C:129.*G1*$^{1.05}$	Surviving
165	132	365	F2 C:129.*G1*$^{1.05}$	Surviving
166	185	365	F2 C:129.*G1*$^{1.05}$	Surviving
167	187	365	F2 C:129.*G1*$^{1.05}$	Surviving
168	201	365	F2 C:129.*G1*$^{1.05}$	Surviving
169	217	365	F2 C:129.*G1*$^{1.05}$	Surviving
170	221	365	F2 C:129.*G1*$^{1.05}$	Surviving
171	247	365	F2 C:129.*G1*$^{1.05}$	Surviving
172	249	365	F2 C:129.*G1*$^{1.05}$	Surviving
173	330	365	F2 C:129.*G1*$^{1.05}$	Surviving
174	367	365	F2 C:129.*G1*$^{1.05}$	Surviving
175	395	365	F2 C:129.*G1*$^{1.05}$	Surviving
176	403	365	F2 C:129.*G1*$^{1.05}$	Surviving
177	421	365	F2 C:129.*G1*$^{1.05}$	Surviving
178	425	365	F2 C:129.*G1*$^{1.05}$	Surviving
179	437	365	F2 C:129.*G1*$^{1.05}$	Surviving
180	495	365	F2 C:129.*G1*$^{1.05}$	Surviving
181	536	365	F2 C:129.*G1*$^{1.05}$	Surviving
182	537	365	F2 C:129.*G1*$^{1.05}$	Surviving
183	549	365	F2 C:129.*G1*$^{1.05}$	Surviving
184	598	365	F2 C:129.*G1*$^{1.05}$	Surviving
185	678	365	F2 C:129.*G1*$^{1.05}$	Surviving
186	687	365	F2 C:129.*G1*$^{1.05}$	Surviving
187	694	365	F2 C:129.*G1*$^{1.05}$	Surviving
188	695	365	F2 C:129.*G1*$^{1.05}$	Surviving
189	719	365	F2 C:129.*G1*$^{1.05}$	Surviving
190	722	365	F2 C:129.*G1*$^{1.05}$	Surviving
191	741	365	F2 C:129.*G1*$^{1.05}$	Surviving
192	744	365	F2 C:129.*G1*$^{1.05}$	Surviving
193	746	365	F2 C:129.*G1*$^{1.05}$	Surviving
194	770	365	F2 C:129.*G1*$^{1.05}$	Surviving
195	778	365	F2 C:129.*G1*$^{1.05}$	Surviving
196	790	365	F2 C:129.*G1*$^{1.05}$	Surviving
197	791	365	F2 C:129.*G1*$^{1.05}$	Surviving
198	840	365	F2 C:129.*G1*$^{1.05}$	Surviving
199	881	365	F2 C:129.*G1*$^{1.05}$	Surviving
200	889	365	F2 C:129.*G1*$^{1.05}$	Surviving

Supplementary Table S3. List of mice used for experiment shown in Figure 2D, F.

*: Mice that underwent necropsy

No.		ID	Age (day)	Background	Outcome
1	*	002	173	N2 B6.$G1^{1.05}$	Leukemia
2	*	013	213	N2 B6.$G1^{1.05}$	Leukemia
3	*	018	110	N2 B6.$G1^{1.05}$	Leukemia
4	*	097	110	N2 B6.$G1^{1.05}$	Leukemia
5	*	099	149	N2 B6.$G1^{1.05}$	Leukemia
6	*	102	174	N2 B6.$G1^{1.05}$	Leukemia
7	*	115	187	N2 B6.$G1^{1.05}$	Leukemia
8	*	139	124	N2 B6.$G1^{1.05}$	Leukemia
9	*	142	143	N2 B6.$G1^{1.05}$	Leukemia
10	*	168	192	N2 B6.$G1^{1.05}$	Leukemia
11	*	174	189	N2 B6.$G1^{1.05}$	Leukemia
12	*	185	250	N2 B6.$G1^{1.05}$	Leukemia
13	*	247	70	N2 B6.$G1^{1.05}$	Leukemia
14	*	291	161	N2 B6.$G1^{1.05}$	Leukemia
15	*	344	221	N2 B6.$G1^{1.05}$	Leukemia
16	*	351	147	N2 B6.$G1^{1.05}$	Leukemia
17	*	397	123	N2 B6.$G1^{1.05}$	Leukemia
18	*	425	102	N2 B6.$G1^{1.05}$	Leukemia
19	*	433	146	N2 B6.$G1^{1.05}$	Leukemia
20	*	443	250	N2 B6.$G1^{1.05}$	Leukemia
21	*	449	122	N2 B6.$G1^{1.05}$	Leukemia
22	*	459	124	N2 B6.$G1^{1.05}$	Leukemia
23	*	467	226	N2 B6.$G1^{1.05}$	Leukemia
24	*	505	223	N2 B6.$G1^{1.05}$	Leukemia
25	*	518	121	N2 B6.$G1^{1.05}$	Leukemia
26	*	550	193	N2 B6.$G1^{1.05}$	Leukemia
27	*	573	205	N2 B6.$G1^{1.05}$	Leukemia
28	*	583	152	N2 B6.$G1^{1.05}$	Leukemia
29	*	605	102	N2 B6.$G1^{1.05}$	Leukemia
30	*	651	170	N2 B6.$G1^{1.05}$	Leukemia
31	*	654	72	N2 B6.$G1^{1.05}$	Leukemia
32	*	656	205	N2 B6.$G1^{1.05}$	Leukemia
33	*	658	121	N2 B6.$G1^{1.05}$	Leukemia
34	*	659	119	N2 B6.$G1^{1.05}$	Leukemia
35	*	824	103	N2 B6.$G1^{1.05}$	Leukemia
36	*	828	161	N2 B6.$G1^{1.05}$	Leukemia
37	*	833	164	N2 B6.$G1^{1.05}$	Leukemia
38	*	837	99	N2 B6.$G1^{1.05}$	Leukemia
39	*	840	87	N2 B6.$G1^{1.05}$	Leukemia
40	*	847	113	N2 B6.$G1^{1.05}$	Leukemia

Supplementary Table S3 (Continued)

No.		ID	Age (day)	Background	Outcome
41	*	861	189	N2 B6.*G1*$^{1.05}$	Leukemia
42	*	867	136	N2 B6.*G1*$^{1.05}$	Leukemia
43	*	872	135	N2 B6.*G1*$^{1.05}$	Leukemia
44	*	874	118	N2 B6.*G1*$^{1.05}$	Leukemia
45	*	875	118	N2 B6.*G1*$^{1.05}$	Leukemia
46	*	001-2	224	N2 B6.*G1*$^{1.05}$	Leukemia
47	*	174	101	N2 B6.*G1*$^{1.05}$	Leukemia
48	*	279	197	N2 B6.*G1*$^{1.05}$	Leukemia
49	*	281	117	N2 B6.*G1*$^{1.05}$	Leukemia
50	*	285	110	N2 B6.*G1*$^{1.05}$	Leukemia
51	*	390	116	N2 B6.*G1*$^{1.05}$	Leukemia
52	*	404	117	N2 B6.*G1*$^{1.05}$	Leukemia
53	*	474	126	N2 B6.*G1*$^{1.05}$	Leukemia
54	*	505	122	N2 B6.*G1*$^{1.05}$	Leukemia
55	*	550	161	N2 B6.*G1*$^{1.05}$	Leukemia
56	*	624	100	N2 B6.*G1*$^{1.05}$	Leukemia
57	*	634	127	N2 B6.*G1*$^{1.05}$	Leukemia
58	*	650-2	205	N2 B6.*G1*$^{1.05}$	Leukemia
59	*	792	93	N2 B6.*G1*$^{1.05}$	Leukemia
60	*	795	93	N2 B6.*G1*$^{1.05}$	Leukemia
61	*	799	106	N2 B6.*G1*$^{1.05}$	Leukemia
62	*	820	140	N2 B6.*G1*$^{1.05}$	Leukemia
63	*	822	153	N2 B6.*G1*$^{1.05}$	Leukemia
64		024	292	N2 B6.*G1*$^{1.05}$	Unknown
65		140	192	N2 B6.*G1*$^{1.05}$	Unknown
66		172	133	N2 B6.*G1*$^{1.05}$	Unknown
67		249	118	N2 B6.*G1*$^{1.05}$	Unknown
68		295	241	N2 B6.*G1*$^{1.05}$	Unknown
69		334	275	N2 B6.*G1*$^{1.05}$	Unknown
70		343	156	N2 B6.*G1*$^{1.05}$	Unknown
71		416	60	N2 B6.*G1*$^{1.05}$	Unknown
72		426	102	N2 B6.*G1*$^{1.05}$	Unknown
73		503	161	N2 B6.*G1*$^{1.05}$	Unknown
74		594	123	N2 B6.*G1*$^{1.05}$	Unknown
75		608	142	N2 B6.*G1*$^{1.05}$	Unknown
76		670	104	N2 B6.*G1*$^{1.05}$	Unknown
77		175	110	N2 B6.*G1*$^{1.05}$	Unknown
78		286	105	N2 B6.*G1*$^{1.05}$	Unknown
79		354	143	N2 B6.*G1*$^{1.05}$	Unknown
80		401	240	N2 B6.*G1*$^{1.05}$	Unknown

Supplementary Table S3 (Continued)

No.		ID	Age (day)	Background	Outcome
81		675	217	N2 B6.*G1*$^{1.05}$	Unknown
82		794	104	N2 B6.*G1*$^{1.05}$	Unknown
83		798	106	N2 B6.*G1*$^{1.05}$	Unknown
84	*	508	297	N2 B6.*G1*$^{1.05}$	Unknown
85	*	758	284	N2 B6.*G1*$^{1.05}$	Unknown
86		003	300	N2 B6.*G1*$^{1.05}$	Surviving
87		004	300	N2 B6.*G1*$^{1.05}$	Surviving
88		006	300	N2 B6.*G1*$^{1.05}$	Surviving
89		011	300	N2 B6.*G1*$^{1.05}$	Surviving
90		012	300	N2 B6.*G1*$^{1.05}$	Surviving
91		014	300	N2 B6.*G1*$^{1.05}$	Surviving
92		015	300	N2 B6.*G1*$^{1.05}$	Surviving
93		017	300	N2 B6.*G1*$^{1.05}$	Surviving
94		020	300	N2 B6.*G1*$^{1.05}$	Surviving
95		021	300	N2 B6.*G1*$^{1.05}$	Surviving
96		038	300	N2 B6.*G1*$^{1.05}$	Surviving
97		042	300	N2 B6.*G1*$^{1.05}$	Surviving
98		043	300	N2 B6.*G1*$^{1.05}$	Surviving
99		044	300	N2 B6.*G1*$^{1.05}$	Surviving
100		081	300	N2 B6.*G1*$^{1.05}$	Surviving
101		090	300	N2 B6.*G1*$^{1.05}$	Surviving
102		092	300	N2 B6.*G1*$^{1.05}$	Surviving
103		096	300	N2 B6.*G1*$^{1.05}$	Surviving
104		101	300	N2 B6.*G1*$^{1.05}$	Surviving
105		103	300	N2 B6.*G1*$^{1.05}$	Surviving
106		105	300	N2 B6.*G1*$^{1.05}$	Surviving
107		107	300	N2 B6.*G1*$^{1.05}$	Surviving
108		109	300	N2 B6.*G1*$^{1.05}$	Surviving
109		116	300	N2 B6.*G1*$^{1.05}$	Surviving
110		119	300	N2 B6.*G1*$^{1.05}$	Surviving
111		120	300	N2 B6.*G1*$^{1.05}$	Surviving
112		122	300	N2 B6.*G1*$^{1.05}$	Surviving
113		124	300	N2 B6.*G1*$^{1.05}$	Surviving
114		128	300	N2 B6.*G1*$^{1.05}$	Surviving
115		133	300	N2 B6.*G1*$^{1.05}$	Surviving
116		137	300	N2 B6.*G1*$^{1.05}$	Surviving
117		141	300	N2 B6.*G1*$^{1.05}$	Surviving
118		142	300	N2 B6.*G1*$^{1.05}$	Surviving
119		143	300	N2 B6.*G1*$^{1.05}$	Surviving
120		167	300	N2 B6.*G1*$^{1.05}$	Surviving

Supplementary Table S3 (Continued)

No.	ID	Age (day)	Background	Outcome
121	173	300	N2 B6.$G1^{1.05}$	Surviving
122	175	300	N2 B6.$G1^{1.05}$	Surviving
123	187	300	N2 B6.$G1^{1.05}$	Surviving
124	188	300	N2 B6.$G1^{1.05}$	Surviving
125	189	300	N2 B6.$G1^{1.05}$	Surviving
126	190	300	N2 B6.$G1^{1.05}$	Surviving
127	242	300	N2 B6.$G1^{1.05}$	Surviving
128	245	300	N2 B6.$G1^{1.05}$	Surviving
129	252	300	N2 B6.$G1^{1.05}$	Surviving
130	253	300	N2 B6.$G1^{1.05}$	Surviving
131	270	300	N2 B6.$G1^{1.05}$	Surviving
132	273	300	N2 B6.$G1^{1.05}$	Surviving
133	275	300	N2 B6.$G1^{1.05}$	Surviving
134	282	300	N2 B6.$G1^{1.05}$	Surviving
135	288	300	N2 B6.$G1^{1.05}$	Surviving
136	290	300	N2 B6.$G1^{1.05}$	Surviving
137	292	300	N2 B6.$G1^{1.05}$	Surviving
138	294	300	N2 B6.$G1^{1.05}$	Surviving
139	296	300	N2 B6.$G1^{1.05}$	Surviving
140	297	300	N2 B6.$G1^{1.05}$	Surviving
141	321	300	N2 B6.$G1^{1.05}$	Surviving
142	324	300	N2 B6.$G1^{1.05}$	Surviving
143	326	300	N2 B6.$G1^{1.05}$	Surviving
144	336	300	N2 B6.$G1^{1.05}$	Surviving
145	337	300	N2 B6.$G1^{1.05}$	Surviving
146	348	300	N2 B6.$G1^{1.05}$	Surviving
147	349	300	N2 B6.$G1^{1.05}$	Surviving
148	350	300	N2 B6.$G1^{1.05}$	Surviving
149	374	300	N2 B6.$G1^{1.05}$	Surviving
150	377	300	N2 B6.$G1^{1.05}$	Surviving
151	382	300	N2 B6.$G1^{1.05}$	Surviving
152	407	300	N2 B6.$G1^{1.05}$	Surviving
153	409	300	N2 B6.$G1^{1.05}$	Surviving
154	410	300	N2 B6.$G1^{1.05}$	Surviving
155	418	300	N2 B6.$G1^{1.05}$	Surviving
156	419	300	N2 B6.$G1^{1.05}$	Surviving
157	420	300	N2 B6.$G1^{1.05}$	Surviving
158	431	300	N2 B6.$G1^{1.05}$	Surviving
159	434	300	N2 B6.$G1^{1.05}$	Surviving
160	442	300	N2 B6.$G1^{1.05}$	Surviving

Supplementary Table S3 (Continued)

No.	ID	Age (day)	Background	Outcome
161	462	300	N2 B6.$G1^{1.05}$	Surviving
162	468	300	N2 B6.$G1^{1.05}$	Surviving
163	469	300	N2 B6.$G1^{1.05}$	Surviving
164	470	300	N2 B6.$G1^{1.05}$	Surviving
165	474	300	N2 B6.$G1^{1.05}$	Surviving
166	478	300	N2 B6.$G1^{1.05}$	Surviving
167	501	300	N2 B6.$G1^{1.05}$	Surviving
168	502	300	N2 B6.$G1^{1.05}$	Surviving
169	509	300	N2 B6.$G1^{1.05}$	Surviving
170	510	300	N2 B6.$G1^{1.05}$	Surviving
171	512	300	N2 B6.$G1^{1.05}$	Surviving
172	513	300	N2 B6.$G1^{1.05}$	Surviving
173	514	300	N2 B6.$G1^{1.05}$	Surviving
174	530	300	N2 B6.$G1^{1.05}$	Surviving
175	547	300	N2 B6.$G1^{1.05}$	Surviving
176	549	300	N2 B6.$G1^{1.05}$	Surviving
177	574	300	N2 B6.$G1^{1.05}$	Surviving
178	576	300	N2 B6.$G1^{1.05}$	Surviving
179	582	300	N2 B6.$G1^{1.05}$	Surviving
180	587	300	N2 B6.$G1^{1.05}$	Surviving
181	595	300	N2 B6.$G1^{1.05}$	Surviving
182	596	300	N2 B6.$G1^{1.05}$	Surviving
183	598	300	N2 B6.$G1^{1.05}$	Surviving
184	601	300	N2 B6.$G1^{1.05}$	Surviving
185	602	300	N2 B6.$G1^{1.05}$	Surviving
186	603	300	N2 B6.$G1^{1.05}$	Surviving
187	604	300	N2 B6.$G1^{1.05}$	Surviving
188	607	300	N2 B6.$G1^{1.05}$	Surviving
189	609	300	N2 B6.$G1^{1.05}$	Surviving
190	627	300	N2 B6.$G1^{1.05}$	Surviving
191	629	300	N2 B6.$G1^{1.05}$	Surviving
192	645	300	N2 B6.$G1^{1.05}$	Surviving
193	646	300	N2 B6.$G1^{1.05}$	Surviving
194	648	300	N2 B6.$G1^{1.05}$	Surviving
195	649	300	N2 B6.$G1^{1.05}$	Surviving
196	650	300	N2 B6.$G1^{1.05}$	Surviving
197	655	300	N2 B6.$G1^{1.05}$	Surviving
198	657	300	N2 B6.$G1^{1.05}$	Surviving
199	664	300	N2 B6.$G1^{1.05}$	Surviving
200	665	300	N2 B6.$G1^{1.05}$	Surviving

Supplementary Table S3 (Continued)

No.	ID	Age (day)	Background	Outcome
201	672	300	N2 B6.$G1^{1.05}$	Surviving
202	676	300	N2 B6.$G1^{1.05}$	Surviving
203	682	300	N2 B6.$G1^{1.05}$	Surviving
204	683	300	N2 B6.$G1^{1.05}$	Surviving
205	687	300	N2 B6.$G1^{1.05}$	Surviving
206	691	300	N2 B6.$G1^{1.05}$	Surviving
207	692	300	N2 B6.$G1^{1.05}$	Surviving
208	693	300	N2 B6.$G1^{1.05}$	Surviving
209	700	300	N2 B6.$G1^{1.05}$	Surviving
210	741	300	N2 B6.$G1^{1.05}$	Surviving
211	743	300	N2 B6.$G1^{1.05}$	Surviving
212	745	300	N2 B6.$G1^{1.05}$	Surviving
213	747	300	N2 B6.$G1^{1.05}$	Surviving
214	751	300	N2 B6.$G1^{1.05}$	Surviving
215	759	300	N2 B6.$G1^{1.05}$	Surviving
216	766	300	N2 B6.$G1^{1.05}$	Surviving
217	771	300	N2 B6.$G1^{1.05}$	Surviving
218	779	300	N2 B6.$G1^{1.05}$	Surviving
219	780	300	N2 B6.$G1^{1.05}$	Surviving
220	814	300	N2 B6.$G1^{1.05}$	Surviving
221	815	300	N2 B6.$G1^{1.05}$	Surviving
222	822	300	N2 B6.$G1^{1.05}$	Surviving
223	834	300	N2 B6.$G1^{1.05}$	Surviving
224	836	300	N2 B6.$G1^{1.05}$	Surviving
225	839	300	N2 B6.$G1^{1.05}$	Surviving
226	842	300	N2 B6.$G1^{1.05}$	Surviving
227	848	300	N2 B6.$G1^{1.05}$	Surviving
228	856	300	N2 B6.$G1^{1.05}$	Surviving
229	860	300	N2 B6.$G1^{1.05}$	Surviving
230	862	300	N2 B6.$G1^{1.05}$	Surviving
231	873	300	N2 B6.$G1^{1.05}$	Surviving
232	877	300	N2 B6.$G1^{1.05}$	Surviving
233	879	300	N2 B6.$G1^{1.05}$	Surviving
234	116-2	300	N2 B6.$G1^{1.05}$	Surviving
235	134-2	300	N2 B6.$G1^{1.05}$	Surviving
236	169-2	300	N2 B6.$G1^{1.05}$	Surviving
237	240	300	N2 B6.$G1^{1.05}$	Surviving
238	243	300	N2 B6.$G1^{1.05}$	Surviving
239	280	300	N2 B6.$G1^{1.05}$	Surviving
240	284	300	N2 B6.$G1^{1.05}$	Surviving

Supplementary Table S3 (Continued)

No.		ID	Age (day)	Background	Outcome
241		291-2	300	N2 B6.*G1*$^{1.05}$	Surviving
242		297-2	300	N2 B6.*G1*$^{1.05}$	Surviving
243		003-2	300	N2 B6.*G1*$^{1.05}$	Surviving
244		337	300	N2 B6.*G1*$^{1.05}$	Surviving
245		398	300	N2 B6.*G1*$^{1.05}$	Surviving
246		399	300	N2 B6.*G1*$^{1.05}$	Surviving
247		004-2	300	N2 B6.*G1*$^{1.05}$	Surviving
248		400-2	300	N2 B6.*G1*$^{1.05}$	Surviving
249		401-2	300	N2 B6.*G1*$^{1.05}$	Surviving
250		402	300	N2 B6.*G1*$^{1.05}$	Surviving
251		403-2	300	N2 B6.*G1*$^{1.05}$	Surviving
252		422-2	300	N2 B6.*G1*$^{1.05}$	Surviving
253		473	300	N2 B6.*G1*$^{1.05}$	Surviving
254		475	300	N2 B6.*G1*$^{1.05}$	Surviving
255		505-2	300	N2 B6.*G1*$^{1.05}$	Surviving
256		510	300	N2 B6.*G1*$^{1.05}$	Surviving
257		510-2	300	N2 B6.*G1*$^{1.05}$	Surviving
258		513	300	N2 B6.*G1*$^{1.05}$	Surviving
259		549-2	300	N2 B6.*G1*$^{1.05}$	Surviving
260		549-2	300	N2 B6.*G1*$^{1.05}$	Surviving
261		551	300	N2 B6.*G1*$^{1.05}$	Surviving
262		6-2	300	N2 B6.*G1*$^{1.05}$	Surviving
263		637	300	N2 B6.*G1*$^{1.05}$	Surviving
264		683-2	300	N2 B6.*G1*$^{1.05}$	Surviving
265	*	170	211	N2 C.*G1*$^{1.05}$	Leukemia
266	*	413	152	N2 C.*G1*$^{1.05}$	Leukemia
267	*	422	158	N2 C.*G1*$^{1.05}$	Leukemia
268		118	137	N2 C.*G1*$^{1.05}$	Unknown
269		162	135	N2 C.*G1*$^{1.05}$	Unknown
270		178	284	N2 C.*G1*$^{1.05}$	Unknown
271		852	132	N2 C.*G1*$^{1.05}$	Unknown
272		899	109	N2 C.*G1*$^{1.05}$	Unknown
273		001	300	N2 C.*G1*$^{1.05}$	Surviving
274		062	300	N2 C.*G1*$^{1.05}$	Surviving
275		110	300	N2 C.*G1*$^{1.05}$	Surviving
276		111	300	N2 C.*G1*$^{1.05}$	Surviving
277		121	300	N2 C.*G1*$^{1.05}$	Surviving
278		148	300	N2 C.*G1*$^{1.05}$	Surviving
279		152	300	N2 C.*G1*$^{1.05}$	Surviving
280		163	300	N2 C.*G1*$^{1.05}$	Surviving

Supplementary Table S3 (Continued)

No.	ID	Age (day)	Background	Outcome
281	172	300	N2 C.*G1*$^{1.05}$	Surviving
282	176	300	N2 C.*G1*$^{1.05}$	Surviving
283	182	300	N2 C.*G1*$^{1.05}$	Surviving
284	184	300	N2 C.*G1*$^{1.05}$	Surviving
285	401	300	N2 C.*G1*$^{1.05}$	Surviving
286	402	300	N2 C.*G1*$^{1.05}$	Surviving
287	403	300	N2 C.*G1*$^{1.05}$	Surviving
288	421	300	N2 C.*G1*$^{1.05}$	Surviving
289	472	300	N2 C.*G1*$^{1.05}$	Surviving
290	528	300	N2 C.*G1*$^{1.05}$	Surviving
291	537	300	N2 C.*G1*$^{1.05}$	Surviving
292	543	300	N2 C.*G1*$^{1.05}$	Surviving
293	562	300	N2 C.*G1*$^{1.05}$	Surviving
294	565	300	N2 C.*G1*$^{1.05}$	Surviving
295	586	300	N2 C.*G1*$^{1.05}$	Surviving
296	588	300	N2 C.*G1*$^{1.05}$	Surviving
297	593	300	N2 C.*G1*$^{1.05}$	Surviving
298	622	300	N2 C.*G1*$^{1.05}$	Surviving
299	639	300	N2 C.*G1*$^{1.05}$	Surviving
300	661	300	N2 C.*G1*$^{1.05}$	Surviving
301	681	300	N2 C.*G1*$^{1.05}$	Surviving
302	706	300	N2 C.*G1*$^{1.05}$	Surviving
303	707	300	N2 C.*G1*$^{1.05}$	Surviving
304	709	300	N2 C.*G1*$^{1.05}$	Surviving
305	843	300	N2 C.*G1*$^{1.05}$	Surviving
306	844	300	N2 C.*G1*$^{1.05}$	Surviving
307	857	300	N2 C.*G1*$^{1.05}$	Surviving
308	858	300	N2 C.*G1*$^{1.05}$	Surviving
309	890	300	N2 C.*G1*$^{1.05}$	Surviving
310	891	300	N2 C.*G1*$^{1.05}$	Surviving
311	894	300	N2 C.*G1*$^{1.05}$	Surviving
312	895	300	N2 C.*G1*$^{1.05}$	Surviving
313	175-2	300	N2 C.*G1*$^{1.05}$	Surviving
314	188-2	300	N2 C.*G1*$^{1.05}$	Surviving
315	402-2	300	N2 C.*G1*$^{1.05}$	Surviving
316	410-2	300	N2 C.*G1*$^{1.05}$	Surviving
317	426-2	300	N2 C.*G1*$^{1.05}$	Surviving
318	470-2	300	N2 C.*G1*$^{1.05}$	Surviving
319	583-2	300	N2 C.*G1*$^{1.05}$	Surviving
320	659-2	300	N2 C.*G1*$^{1.05}$	Surviving

Supplementary Table S3 (Continued)

No.		ID	Age (day)	Background	Outcome
321		682-2	300	N2 C.*G1*$^{1.05}$	Surviving
322	*	123	147	F1 C:B6.*G1*$^{1.05}$	Leukemia
323	*	125	206	F1 C:B6.*G1*$^{1.05}$	Leukemia
324	*	236	108	F1 C:B6.*G1*$^{1.05}$	Leukemia
325		234	111	F1 C:B6.*G1*$^{1.05}$	Unknown
326		25	300	F1 C:B6.*G1*$^{1.05}$	Surviving
327		26	300	F1 C:B6.*G1*$^{1.05}$	Surviving
328		57	300	F1 C:B6.*G1*$^{1.05}$	Surviving
329		59	300	F1 C:B6.*G1*$^{1.05}$	Surviving
330		60	300	F1 C:B6.*G1*$^{1.05}$	Surviving
331		64	300	F1 C:B6.*G1*$^{1.05}$	Surviving
332		127	300	F1 C:B6.*G1*$^{1.05}$	Surviving
333		132	300	F1 C:B6.*G1*$^{1.05}$	Surviving
334		134	300	F1 C:B6.*G1*$^{1.05}$	Surviving
335		177	300	F1 C:B6.*G1*$^{1.05}$	Surviving
336		381	300	F1 C:B6.*G1*$^{1.05}$	Surviving
337		540	300	F1 C:B6.*G1*$^{1.05}$	Surviving
338		541	300	F1 C:B6.*G1*$^{1.05}$	Surviving
339		589	300	F1 C:B6.*G1*$^{1.05}$	Surviving
340		641	300	F1 C:B6.*G1*$^{1.05}$	Surviving
341		643	300	F1 C:B6.*G1*$^{1.05}$	Surviving
342		704	300	F1 C:B6.*G1*$^{1.05}$	Surviving
343		753	300	F1 C:B6.*G1*$^{1.05}$	Surviving
344		754	300	F1 C:B6.*G1*$^{1.05}$	Surviving
345		757	300	F1 C:B6.*G1*$^{1.05}$	Surviving
346		014	300	F1 C:B6.*G1*$^{1.05}$	Surviving
347		015	300	F1 C:B6.*G1*$^{1.05}$	Surviving
348		054	300	F1 C:B6.*G1*$^{1.05}$	Surviving
349		176-2	300	F1 C:B6.*G1*$^{1.05}$	Surviving
350		182-2	300	F1 C:B6.*G1*$^{1.05}$	Surviving
351		382-2	300	F1 C:B6.*G1*$^{1.05}$	Surviving

Supplementary information

Materials and Methods

Mice

Experimental procedures are approved by the Institutional Animal Experiment Committee of the Tohoku University (2018MdA-058, 2019mdA-289). *Gata1.05*/X mice were bred in-house at the animal facility in Tohoku University. Wild-type of mice were purchased from supplier (Kumagai-Shigeyasu Co., Ltd). Mice having enlarged spleen over the midline of body at necropsy were defined as those developing leukemia. Peripheral blood samples were collected from submandibular vein under anesthesia with isoflurane, subsequently the mice were euthanized for the tissue sample collection.

Histological, cytospin and flow cytometry analyses

For histological examination, spleens were fixed by Mildform® 10N (FUJIFILM). Sections of formalin-fixed and paraffin-embed tissue samples were stained by hematoxylin (Merck) and eosin (Merck) staining solution. Cytospin samples of splenic cells were prepared by cytocentrifuge (Thermo Fisher Scientific) and stained with May–Grünwald staining solution (Merck) and Giemsa staining solution (Merck). For flow cytometry assay, bone marrow cells were stained with allophycocyanin-conjugated anti-c-Kit and fluorescein isothiocyanate-conjugated anti-CD71 antibodies (BD Pharmingen). The samples were subjected to flow cytometry analyses using FACSVerse (BD Biosciences). Data were analyzed with FlowJo (TreeStar) software.

Gene expression analysis

Total tissue RNA was extracted using Isogen (Nippon-Gene). cDNA was synthesized using a ReverTra Ace qPCR RT kit (Toyobo). qPCR was performed using a StepOne plus (Applied Biosystems) with target-specific primers and Thunderbird qPCR mix (Toyobo). The sequence information for all primers used in the qPCR analysis are as follow; *Gata1* (5-cagaaccggcctctcatcc and 5-tagtgcattgggtgcctgc); *Hprt* (5-gttggatacaggccagactttgt andccacaggactagaacacctgc).

Statistical analysis

All statistical analyses were done using JMP software (SAS Institute Inc.). Survival rates were analyzed using the Kaplan-Meier method. For comparisons among five groups, the Kruskal–Wallis test was performed to evaluate differences among the five groups. If the p value was less than 0.05, Steel-Dwass multiple comparisons were further performed to evaluate differences. Data were considered statistically significant at $P<0.05$.

Accession codes

Gata1 cDNA sequences of 129S1/SvlmJ (MGP_129S1SvImJ_T0095571.1), BALB/cJ (MGP_BALBcJ_T0095579.1), C3H/HeJ (MGP_C3HHeJ_T0095138.1), C57BL/6 (ENSMUST00000033502.14), and DBA/2J (MGP_DBA2J_T0095267.1) strains available from the Ensemble Genome Browser (https://asia.ensembl.org/index.html).
